# Balancing the stability and drug activation in adaptive nanoparticles potentiates chemotherapy in multidrug-resistant cancer

**DOI:** 10.7150/thno.54066

**Published:** 2021-02-19

**Authors:** Jianqin Wan, Lingling Huang, Jiangting Cheng, Huangfu Qi, Jiahui Jin, Hangxiang Wang

**Affiliations:** 1 The First Affiliated Hospital, Zhejiang University School of Medicine; NHC Key Laboratory of Combined Multi-Organ Transplantation; Key Laboratory of Organ Transplantation, Research Center for Diagnosis and Treatment of Hepatobiliary Diseases, Zhejiang Province, Hangzhou, P. R. China.; 2 Institute of Pharmaceutics, College of Pharmaceutical Sciences, Zhejiang University, Hangzhou, Zhejiang 310058, P. R. China.; 3 Xingzhi College, Zhejiang Normal University, Jinhua, Zhejiang 321004, P. R. China.

**Keywords:** cabazitaxel, polyprodrug, adaptive nanoformulation, drug toxicity, nanoparticle delivery

## Abstract

**Rationale:** Prodrug strategies that render the drug temporarily inactive through a cleavable linkage are able to modulate the physicochemical properties of drugs for adaptive nanoparticle (NP) formulation. Here we used cabazitaxel as a model compound to test the validity of our “balancing NP stability and specific drug activation” strategy.

**Methods:** Cabazitaxel is conjugated to hydrophobic polylactide fragments with varying chain lengths *via* a self-immolation linkage, yielding polymeric prodrugs that can be reactivated by reductive agents in cells. Following a nanoprecipitation protocol, cabazitaxel prodrugs can be stably entrapped in amphiphilic polyethylene-*block*-polylactide matrices to form core-shell nanotherapies with augmented colloidal stability.

**Results:** Upon cellular uptake followed by intracellular reduction, the NPs spontaneously release chemically unmodified cabazitaxel and exert high cytotoxicity. Studies with near-infrared dye-labeled NPs demonstrate that the nanodelivery of the prodrugs extends their systemic circulation, accompanied with increased drug concentrations at target tumor sites. In preclinical mouse xenograft models, including two paclitaxel-resistant xenograft models, the nanotherapy shows a remarkably higher efficacy in tumor suppression and an improved safety profile than free cabazitaxel.

**Conclusion:** Collectively, our approach enables more effective and less toxic delivery of the cabazitaxel drug, which could be a new generalizable strategy for re-engineering other toxic and water-insoluble therapeutics.

## Introduction

Taxane-based chemotherapy (e.g., paclitaxel and docetaxel) ranks among the most extensively used drug therapies for many types of cancer. This class of anticancer agents renders tubulin polymerization and arrests dividing cancer cells at the G2/M phase, eventually leading to apoptotic cell death 1, 2. The clinical outcomes, however, have been greatly compromised by dose-limiting toxicities and inherent or acquired resistance 3, 4. These limitations have stressed the need for new taxane candidates. Cabazitaxel, a new semisynthetic taxane, poses such an interest because of its ability to overcome drug resistance arising from current taxane-based paradigms 5. In a phase III TROPIC study, cabazitaxel prolonged the overall survival in patients with metastatic castration-resistant prostate cancer (mCRPC) that are refractory to docetaxel therapy 6-8. Despite these promising anticancer activities, clinical trials have uncovered the high toxicity of cabazitaxel in patients 9. In addition, cabazitaxel in the present clinical formulation (i.e., Jevtana^®^) shows suboptimal pharmacokinetic properties and rapid elimination from the bloodstream after intravenous administration 10. These obstacles limit the activity of Jevtana^®^ and have spurred the development of clinically relevant delivery platforms to expand drug availability for cancer therapy.

Polymeric nanoparticles (NPs) comprising synthetic aliphatic (co)polyesters such as polylactide (PLA), poly(glycolic acid)-polylactide (PLGA), and poly(caprolactone) (PCL) are widely successful for pharmaceutical delivery of active compounds 11, 12. To date, several anticancer nanoparticles made of these matrices have garnered the US Food and Drug Administration (FDA) approval for human use, and more have entered clinical trials 13. Notable advantages are included within these vehicles, including i) biodegradable properties *in vivo*, ii) enhanced solubility for hydrophobic therapeutics, iii) excellent stability in the blood circulation due to a low critical micelle concentration, iv) preferential particle accumulation at the site of interest, such as tumors, v) improved pharmacokinetic properties, and vi) alleviated systemic toxicity with increased drug tolerability. In these scaffolds, drug cargoes are stably entrapped in NPs to avoid premature release during systemic circulation, while spontaneous release of active drugs at tumor sites is necessitated to achieve sufficiently high drug concentrations 14. Consequently, the balance between the stability in the blood and rapid drug activation in diseased sites is among the critical factors for the construction of more effective, less toxic therapeutic nanoparticles.

Prodrug strategies that render the drug temporarily inactive through a cleavable linkage are able to alter the physicochemical properties of drugs 15-17. By appropriately selecting both the modifiers and linker chemistry, the overall prodrugs can be rationally tailored for adaptive nanoparticle formulations 18, 19. With re-engineered architectures, the affinity of the prodrug entities to the nanocarriers can be substantially augmented, while the release rate of drugs responding to the tumor microenvironment can be controlled by altering the linkages between the drugs and the modifiers 20, 21. Therefore, the toxicity of chemotherapies can be alleviated by reducing systemic drug exposure during the blood circulation, and drugs can be injected at higher doses.

**Figure 1:**
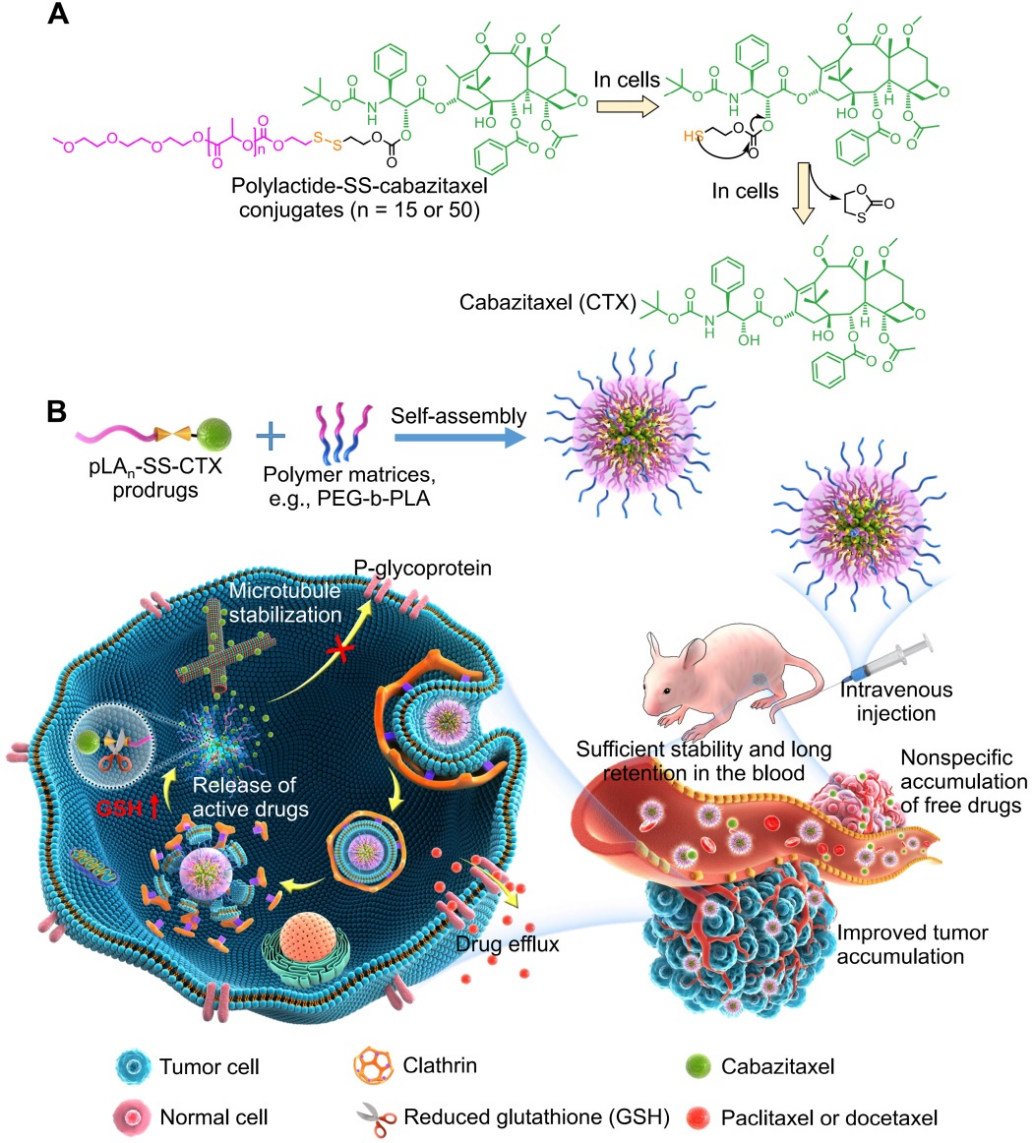
** A**) Chemical structure of polylactide-SS-cabazitaxel conjugates (termed pLA_n_-SS-CTX) and drug activation in cells in response to reduced glutathione (GSH). B) Schematic illustration of self-assembly of prodrugs with amphiphilic copolymers for tumor-specific and on-demand delivery of hydrophobic cabazitaxel agent and systemic treatment of taxane-resistant cancer. Following intravenous injection, the nanomedicines are sufficiently stable to prevent premature release of toxic drugs and to reduce systemic toxicity. Once accumulated at tumor sites *via* passive targeting, nanoparticles are internalized by cancer cells *via* clathrin-mediated endocytosis. Finally, the particles release pharmacologically active cabazitaxel through the cleavage of the disulfide bond in cells, which spontaneously induces cell apoptosis.

Here we describe the role of prodrug reconstitution in controlling the potency of the toxic drug cabazitaxel *via* tuning the balance between nanoparticle stability and intracellular drug activation. To enhance the drug compatibility with the encapsulation matrices and to reduce premature drug release, we molecularly edited cabazitaxel with polylactide segments to generate pLA_n_-SS-CTX prodrugs. In these prodrug constructs, we devised the disulfide linkage to control drug activation in response to intracellular reductive glutathione (GSH) 22. In preclinical mouse models, including paclitaxel-resistant cervical cancer and lung carcinoma models, the administration of pLA_n_-SS-CTX-loaded NPs induced substantial tumor recession, with increased safety profiles relative to the free drug form and free drug-loaded NPs (**Figure 1**).

## Materials and Methods

### Synthesis of pLA_n_-SS-CTX conjugates

pLA_n_-SS-CTX conjugates were synthesized and characterized *via* ^1^H and ^13^C NMR. The synthetic protocols were described in detail in the supporting information.

### Preparation of pLA_n_-SS-CTX prodrug-loaded nanoparticles (pLA_n_-SS-CTX NPs)

pLA_n_-SS-CTX NPs were prepared with a nanoprecipitation method. Briefly, pLA_n_-SS-CTX prodrugs (n=15 or 50) and mPEG_5K_-PLA_8K_ were dissolved in 3 mL of acetone at a weight ratio of 1:19 (at a cabazitaxel equivalence). Then, the solution was added dropwise into 10 mL of DI water under stirring. Following 10 min of stirring, acetone was removed in a rotary evaporator under vacuum. The cabazitaxel concentration of the resulting nanoparticles was analyzed with reversed-phase high performance liquid chromatography (RP-HPLC).

### GSH-responsive hydrolysis of prodrugs

Prodrugs dissolved in 1 mL of DMSO were diluted into DI water containing 10 mM DTT. The mixed solution was incubated at 37 °C under shaking. Prodrugs diluted into DI water without DTT was included as a reference. At predetermined time intervals, samples were collected and analyzed on RP-HPLC equipped with a C8 reversed-phase column at a flow rate of 1.0 mL/minute. The gradient for analysis was 30% to 100% acetonitrile in water for 20 min.

### *In vitro* drug release

To assess the redox-responsive release kinetics of pLA_n_-SS-CTX NPs, 3 mL of pLA_n_-SS-CTX NPs (0.1 mg/mL, a cabazitaxel-equivalent concentration) was dialyzed against PBS buffer (20 mL, pH 7.4, 0.3% polysorbate 80) with or without 10 mM DTT. The *in vitro* drug release assay was performed at 37 °C with shaking. Samples were collected at pre-determined times and then, the same volume of PBS buffer or PBS buffer containing 10 mM DTT was supplemented. To simplify the quantification, all samples were incubated with 0.1 M sodium hydroxide at 37 °C for 2 h, and then neutralized with 0.1 M hydrochloric acid. Finally, samples were analyzed with RP-HPLC.

### Cell lines and cell cultures

Human lung cancer A549, cervical cancer HeLa and prostate cancer DU145 cell lines were purchased from the cell bank of the Chinese Academy of Sciences (Shanghai, China). A549, A549/PTX, HeLa, and HeLa/PTX cells were cultured in RPMI-1640, while DU145 cells were maintained in DMEM. All media were supplemented with 10% fetal bovine serum (FBS), penicillin (100 units/mL), and streptomycin (100 μg/mL). Furthermore, all cells were incubated in atmosphere with 5% CO_2_ at 37 °C.

### Immunofluorescence staining assay

HeLa/PTX cells were seeded into a glass-bottom dish at a density of 6×10^4^ cells per well and cultured overnight. Following 48 h incubation with free cabazitaxel and pLA_n_-SS-CTX NPs (at a CTX- equivalent concentration of 15 nM), cells were fixed with 4% paraformaldehyde for 30 min and permeated with 0.5% Triton X-100 for 1.5 h at room temperature. After wash with PBS, cells were blocked with bovine serum albumin for 1 h and subsequently immunostained with acetyl-α-tubulin antibody (Cell Signaling Technology Inc, USA) at 4 °C overnight. Cells were then incubated with Alexa Fluor 555 donkey anti-rabbit (Life Technologies, USA) at room temperature for 2 h and the nuclei were stained with Hoechst 33342 for 15 min. Finally, cells were imaged on a fluorescence microscope (Olympus).

### Cellular uptake mechanism for pLA_n_-SS-CTX NPs in HeLa/PTX cells

HeLa/PTX cells were seeded into 6-well plates at a density of 2×10^5^ cells per well and cultured at 37 °C overnight. To verify that the endocytosis of NPs was energy-dependent, cells were treated with DiI loaded pLA_n_-SS-CTX NPs at 37 °C and 4 °C for 4 h. Cells incubated with fresh medium were included as a reference. Subsequently, cells were harvested and rinsed with PBS. Upon complete washes, cellular uptake was analyzed with flow cytometry. To investigate the possible endocytosis pathway for pLA_n_-SS-CTX NPs, cells were pre-incubated with specific endocytosis inhibitors at 37 °C, for instance, chlorpromazine (10 µg/mL), cytochalasin D (40 µM), filipin III (5 µg/mL)^23^, ^24^. Following 30 min of incubation, DiI loaded pLA_n_-SS-CTX NPs were added to cells and incubated for another 4 h. Cells were harvested and washed with PBS three times. Finally, the cellular uptake was analyzed with flow cytometry.

### Intracellular distribution

The subcellular distribution of NPs was investigated with confocal laser fluorescence microscopy (CLSM). HeLa/PTX cells were seeded into a glass-bottom dish and cultured overnight. Following 1, 2, 4, 6, 8 h incubation with DiI loaded pLA_n_-SS-CTX NPs, cells were washed with PBS three times and subsequently stained with LysoTracker green NDN-26 and Hoechst 33342 at 37 °C for 30 min. After brief washed, cells were observed on CLSM (Olympus IX81-FV3000, Japan).

### Western blotting

For examining the expression of cabazitaxel-related biomarkers, DU145 and HeLa/PTX cells were treated with free cabazitaxel, pLA_15_-SS-CTX NPs and pLA_50_-SS-CTX NPs (at a cabazitaxel-equivalent concentration, 10 nM for DU145 cells and 25 nM for HeLa/PTX cells) for 48 h. Subsequently, cell protein extracts were collected by lysing cells with RIPA buffer. Cell protein extracts for A549, A549/PTX, HeLa, and HeLa/PTX cells without drug treatment were also collected to determine the expression of P-glycoprotein (P-gp) which is coded by ABCB1. All samples were fractionated by 10% SDS-PAGE gels and transferred to polyvinylidene difluoride membranes. Following 1 h blocking in 5% milk in Tris-bufferred saline/0.1% Tween-20 (TBST) at room temperature, membranes were incubated with anti-acetyl-α-tubulin, anti-cleaved caspase 9, anti-cleaved PARP, anti-ABCB1 and anti-β-actin primary antibodies at 4 °C overnight. Upon complete washes with TBST, membranes were further incubated with secondary antibodies for 2 h at room temperature. Subsequently, proteins were investigated using enhanced chemiluminescence system (Bio-Rad, USA).

### *In vivo* biodistribution with pLA_n_-SS-CTX NPs

The *in vivo* biodistribution of pLA_n_-SS-CTX NPs was evaluated in Balb/c nude mice bearing HeLa/PTX tumor xenografts. When the tumor volume reached 80 m^3^, mice were intravenously injected with free DiR (formulated in polysorbate 80/ethanol, 1:1, v/v) and DiR loaded pLA_n_-SS-CTX NPs (termed DiR@NPs) at a DiR-equivalent dose of 1 mg/kg. Whole-body fluorescence imaging was performed with an *in vivo* imaging system at predetermined times (Clairvivo OPT, SHIMADZU Corporation, Kyoto, Japan). At 24 h post-administration, mice were sacrificed, tumors and major organs (heart, liver, spleen, lung, kidney and brain) were collected for *ex vivo* imaging. Then, frozen sections of tumors were prepared and stained with 4,6-diamidino-2-phenylindole (DAPI). The distribution of DiR@NPs in tumors was investigated using CLSM. The tumor targeting capability for pLA_n_-SS-CTX NPs was assessed in two Balb/c nude mice bearing DU145 and A549/PTX tumor xenografts. Free cabazitaxel (formulated in polysorbate 80/ethanol, 1:1, v/v) and pLA_n_-SS-CTX NPs (25 mg/kg, equivalent to free cabazitaxel) were administered *via* the tail vein. At 24 h post-injection, tumors were excised and ground using a high-speed tissue homogenizer (Servicebio, KZ-II). Subsequently, suspensions of tumors were incubated with DTT dissolved in acetonitrile (at a final concentration of 10 mM) for 2 h at 37 °C. Following centrifugation at 12,000 rpm for 10 min, the released cabazitaxel in the supernatant was analyzed with RP-HPLC.

### *In vivo* antitumor activity

The *in vivo* potency of pLA_n_-SS-CTX NPs was evaluated with two Balb/c nude mice bearing tumor xenografts, including DU145 prostate tumors and HeLa/PTX cervical tumors. When the tumor volume reached approximately 100 mm^3^, mice were randomly divided into four groups (n=5 in each group). Mice were injected with saline, free cabazitaxel and pLA_n_-SS-CTX NPs at a cabazitaxel-equivalent dose of 6 mg/kg via the tail vein. Notably, mice bearing DU145 tumor xenografts were administered on days 0, 3, and 6, while mice bearing Hela/PTX cervical tumor xenografts were administered on days 0, 2, and 4. Tumor volumes and body weight were recorded every three days. The tumor volumes were calculated using the following formula: V = (L × W^2^)/2, L: length, W: width, W is smaller than L. At the end of observation, mice were sacrificed and tumors were excised for imaging.

### Assessment of *in vivo* toxicity

The *in vivo* toxicity of pLA_15_-SS-CTX NPs was evaluated and compared with free cabazitaxel in healthy ICR mice (20-25 g). Mice were intravenously injected with free cabazitaxel and pLA_15_-SS-CTX NPs at 12 mg/kg (cabazitaxel-equivalent dose) on days 0, 3, and 6, while mice receiving saline were included as a control. Body weight was recorded for 23 days, while white blood cell count and lymphocyte count were recorded for 15 days. On day 23, mice were sacrificed and major organs, including the heart, liver, spleen, lung and kidney were collected for hematoxylin and eosin (H&E) staining.

### Immunohistochemistry analysis

Excised tumors and major organs were fixed with 4% formaldehyde, embedded in paraffin, and sectioned into 5-µm slices for active caspase-3 and Ki67 detection assays with anti-cleaved-caspase3 and anti-Ki67 (Servicebio). Sections were further stained with hematoxylin and eosin (H&E, Sigma). The stained slices were observed and imaged on a microscope (Olympus, IX71).

### Statistical analysis

All data are presented as the means ± standard deviation (SD). Student's *t*-test was employed to perform statistical analysis. *p* < 0.05 indicates statistical significance (*), *p* < 0.01 indicates high significance (**), and *p* < 0.001 indicates remarkable significance (***).

## Results

### Synthesis and characterization of pLA_n_-SS-CTX conjugates

**Figure 2:**
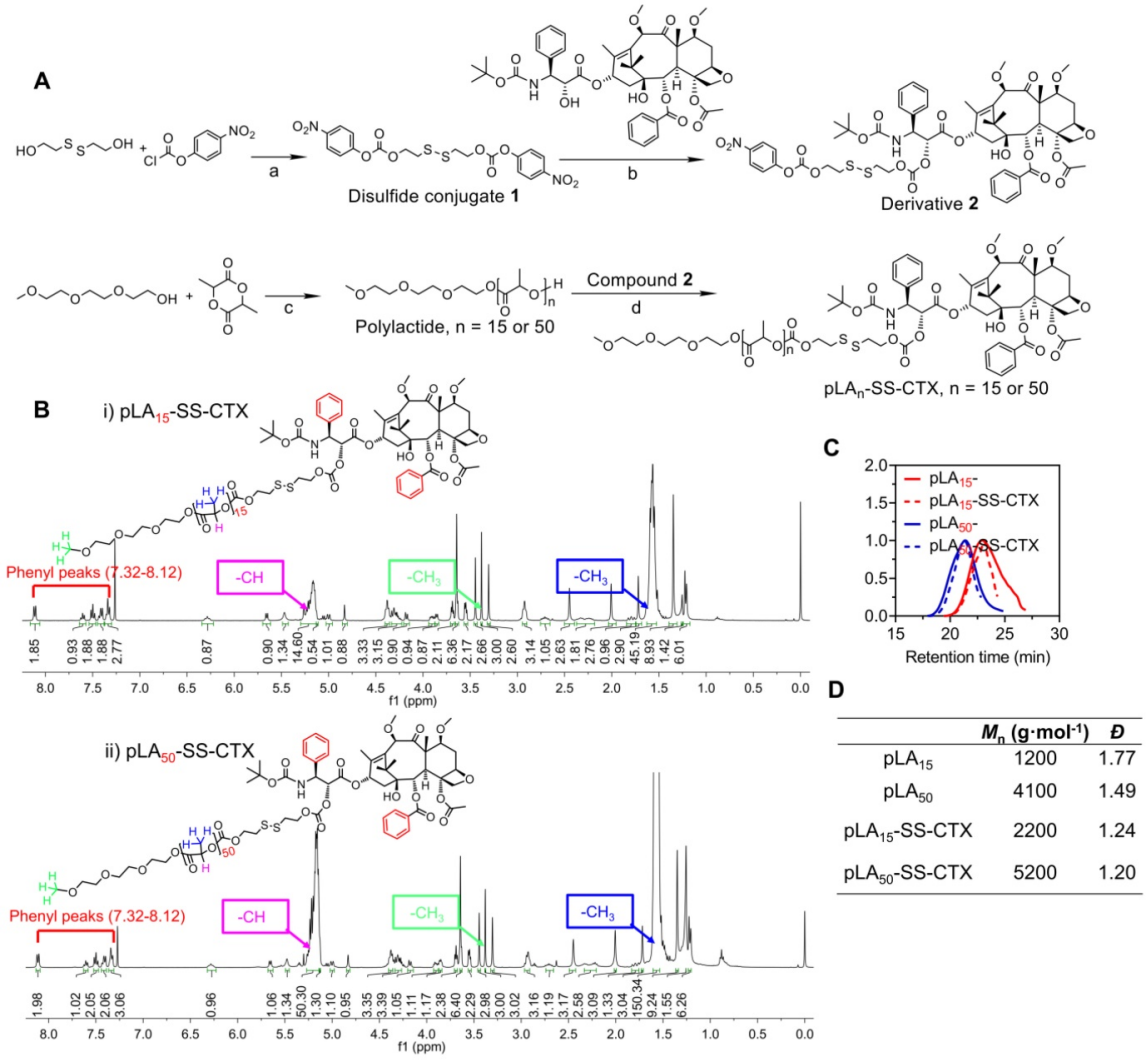
** A**) Synthetic scheme of pLA_n_-SS-CTX conjugates (n = 15 or 50). a) DIEA, DCM, reflux; b) DMAP, DCM, reflux; c) Sn(Oct)_2_, toluene, 140 °C; d) DMAP, DCM, reflux. **B**) ^1^H NMR spectra of pLA_15_-SS-CTX (i) and pLA_50_-SS-CTX (ii) conjugates measured in CDCl_3_. **C**-**D**) Characterization of polylactides (pLA_15_ and pLA_50_) and pLA-drug conjugates using GPC measurements in THF. *M*_n_, number-average molar mass; *Đ*, dispersity.

For the design of a reduction- responsive linker, we integrated a disulfide bond into the polymer-drug conjugates to accomplish drug activation. Reduced GSH is abundant in cells, and upon cleavage of the disulfide bond, a self-immolative elimination reaction takes place to release cytotoxic cabazitaxel in its native form. Specifically, cyclization is rapid and the thiol group can spontaneously cyclize into the proximate carbonyl group (**Figure 1A**) 25. The convergent synthesis route for the prodrugs which we abbreviated as pLA_n_-SS-CTX (n: number of lactide units), is presented in **Figure 2A.** First, disulfide conjugate **1** was synthesized by a base-catalyzed alcoholysis of 4-nitrophenyl chloroformate and bis(2-hydroxyethyl) disulfide. Next, conjugate **1** was reacted with one cabazitaxel molecule, yielding derivative **2**. Finally, **2** was coupled with hydroxyl-terminated polylactides with varying molecular weights to yield the final conjugate pLA_n_-SS-CTX (n = 15 or 50), which was purified by silica gel chromatography. Characterization with the nuclear magnetic resonance (^1^H and ^13^C NMR) spectra verified the successful synthesis of the desired adducts (**Figure 2B** and**Figure S1-8**). The phenyl peaks derived from the attached cabazitaxel molecules at 7.32-8.12 ppm and characteristic peaks for polylactides (5.15-5.26 ppm for -OCH, 3.38 ppm for -CH_3_, 1.55-1.59 ppm for -OCH_3_) were clearly observed in the ^1^H NMR spectra. Moreover, the corresponding peaks (e.g., 126.5-139.5 ppm for aromatic rings, 169.2-170.1 ppm for -OCO-, 69.0-69.3 ppm for -OCH, and 16.7-16.8 ppm for -CH_3_) in ^13^C NMR spectra further confirmed the formation of the pLA_n_-SS-CTX conjugates. Analysis using high-performance liquid chromatography (HPLC) showed the absence of unconjugated cabazitaxel in purified conjugates. The prodrug conjugates were also confirmed with gel-permeation chromatography (GPC) analysis, presenting monomodal mass-distributions (**Figure 2C-D**). These data confirmed that we can readily obtain highly pure pLA_n_-SS-CTX conjugates without the need for tedious synthetic protocols, and nearly one cabazitaxel molecule was attached to each polylactide chain.

### Preparation and characterization of pLA_n_-SS-CTX-loaded NPs

**Figure 3:**
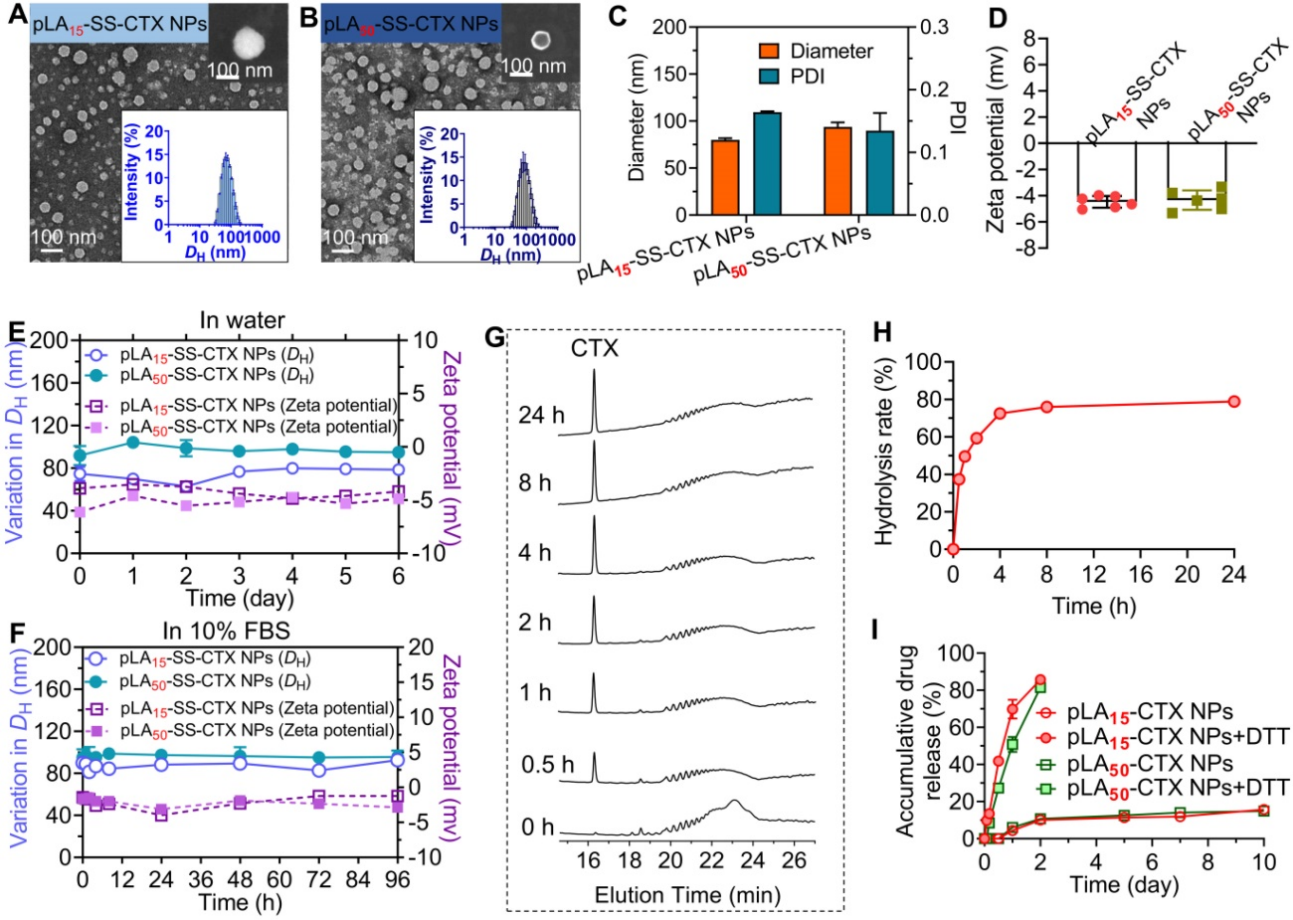
Characterization of pLA_n_-SS-CTX-loaded nanoparticles (n=15 or 50, number of lactide repeating units). Transmission electron microscopy (TEM) images of (**A**) pLA_15_- and (**B**) pLA_50_-SS-CTX NPs. Insert: Scanning electron microscopy (SEM) images and size distribution of pLA_n_-SS-CTX NPs. Scale bars, 100 nm. (**C**) The hydrodynamic diameters (*D*_H_) and (**D**) zeta potential of pLA_n_-SS-CTX NPs measured by dynamic light scattering (DLS). The variations in *D*_H_ (nm) and zeta potential were used to indicate the stability of pLA_n_-SS-CTX NPs in DI water (**E**) and in water containing 10% FBS (**F**) at 37 °C. (**G**) Reduction-responsive degradation of the pLA_15_-SS-CTX prodrug upon treatment with 10 mM DTT analyzed by HPLC. (**H**) Release profile of cabazitaxel from the pLA_15_-SS-CTX prodrug, calculated from the peak intensity at 16.36 min shown in **G**. (**I**) Accumulative drug release profiles from pLA_n_-SS-CTX NPs incubated with or without 10 mM DTT at 37 °C.

The prodrug-loaded NPs which we abbreviated as pLA_n_-SS-CTX NPs were fabricated using a nanoprecipitation method 26. Hereto, the prodrugs were dissolved in acetone and blended with polymer matrices (i.e., PEG_5k_-*b*-PLA_8k_) at a mass ratio of pure drugs to total materials of 1:19 to ensure the drug loading at approximately 5%. The mixture was added dropwise to deionized (DI) water under stirring, and the organic solvent was removed by evaporation. The resultant NPs achieved high drug encapsulation efficiency, with 95.10%±1.00% and 96.91%±1.07% for pLA_15_-SS-CTX and pLA_50_-SS-CTX NPs, respectively. In addition, the drug loadings were determined to be 4.33%±0.05% for pLA_15_-SS-CTX NPs and 3.91%±0.04% for pLA_50_-SS-CTX NPs (**Table 1**). Dynamic light scattering (DLS) measurements revealed that the mean hydrodynamic diameters (*D*_H_) were 80 and 90 nm for pLA_15_-SS-CTX NPs and pLA_50_-SS-CTX NPs, respectively (**Figure 3A-C**). To study the morphologies of therapeutic NPs, transmission electron microscopy (TEM) and scanning electron microscopy (SEM) were used. **Figure 3A-B** showed that both pLA_n_-SS-CTX NPs were spherical in shape with uniform and small sub-100 nm sizes. Such particle sizes could guarantee passive tumor targeting. In addition, slightly negative surface charges were observed for both NPs (i.e., -4.47±0.45 for pLA_15_-SS-CTX NPs and -4.34±0.74 mV for pLA_50_-SS-CTX NPs, **Figure 3D**), which would favor stability for intravenous injection. The nanotherapies exhibited excellent stabilities in water or in 10% FBS-supplemented water, without observed variations in diameters and ζ-potentials for several days (**Figure 3E-F**).

Critical micelle concentration (CMC) was regarded as a key parameter for polymeric NPs to preserve intact in the blood circulation. Therefore, we evaluated the CMC values of pLA_n_-SS-CTX NPs by measuring the intensity of scattered light (**Figure S10**). The CMCs were 13.7 µg/mL for pLA_15_-SS-CTX NPs and 12.7 µg/mL for pLA_50_-SS-CTX NPs, which are sufficiently low for systemic administration (i.e., the estimated concentrations in the blood were 1317.6 µg/mL for pLA_15_-SS-CTX NPs and 1486.2 µg/mL for pLA_50_-SS-CTX NPs). Hence, pLA_n_-SS-CTX NPs are expected to maintain the overall nanostructure intact after intravenous administration.

Owing to the incorporated self-immolative linker in the prodrug constructs, we therefore investigated reduction-responsive drug activation by choosing the pLA_15_-SS-CTX prodrug as a model compound. After the prodrug was exposed to dithiothreitol (DTT, 10 mM), hydrolysis to active cabazitaxel was monitored by HPLC analysis. Following incubation with DTT for 0.5 h, a well-defined peak corresponding to free cabazitaxel occurred (**Figure 3G**). Cabazitaxel release almost plateaued within 4 h, enabling the majority of drugs hydrolyzed from the prodrugs (**Figure 3H**). In sharp contrast, the prodrug was sufficiently stable without drug activation in the absence of DTT (**Figure S9**). We further assessed the drug release kinetics when the conjugate was formulated in polymeric NPs composed of PEG-*b*-PLA copolymer. A dialyzing method was performed against PBS containing DTT at 37 °C. In addition to free cabazitaxel, a series of short oligolactide-tethered drug conjugates were also released from NPs, which complicated the analysis. Hence, the released samples were subjected to the treatment with sodium hydroxide to entirely hydrolyze the compositions according to our previously established protocol 27. The final hydrolytic product (i.e., benzoic acid) was used to quantify the amounts of cabazitaxel. As presented in **Figure 3I**, both pLA_n_-SS-CTX NPs were sufficiently stable without DTT and showed comparable release profiles, with only ~10% cabazitaxel released after the 10-day period. Interestingly, pLA_n_-SS-CTX NPs responded to DTT and rapidly liberated the covalently attached drugs. Compared with pLA_50_-SS-CTX NPs, pLA_15_-SS-CTX NPs showed faster drug release during the initial 24-h period. These results suggest that the chain length of polylactides as modifiers for drug reconstitution greatly impacts the *in vitro* release performance 28. Among the two prodrug constructs, pLA_15_-SS-CTX NPs are expected to show excellent stability during circulation but are more sensitive to intracellular reductive conditions where rapidly released drugs could exert more potent cytotoxic activity.

### Potent *in vitro* cytotoxicity of NPs endowed by reduction-triggered drug release

**Figure 4:**
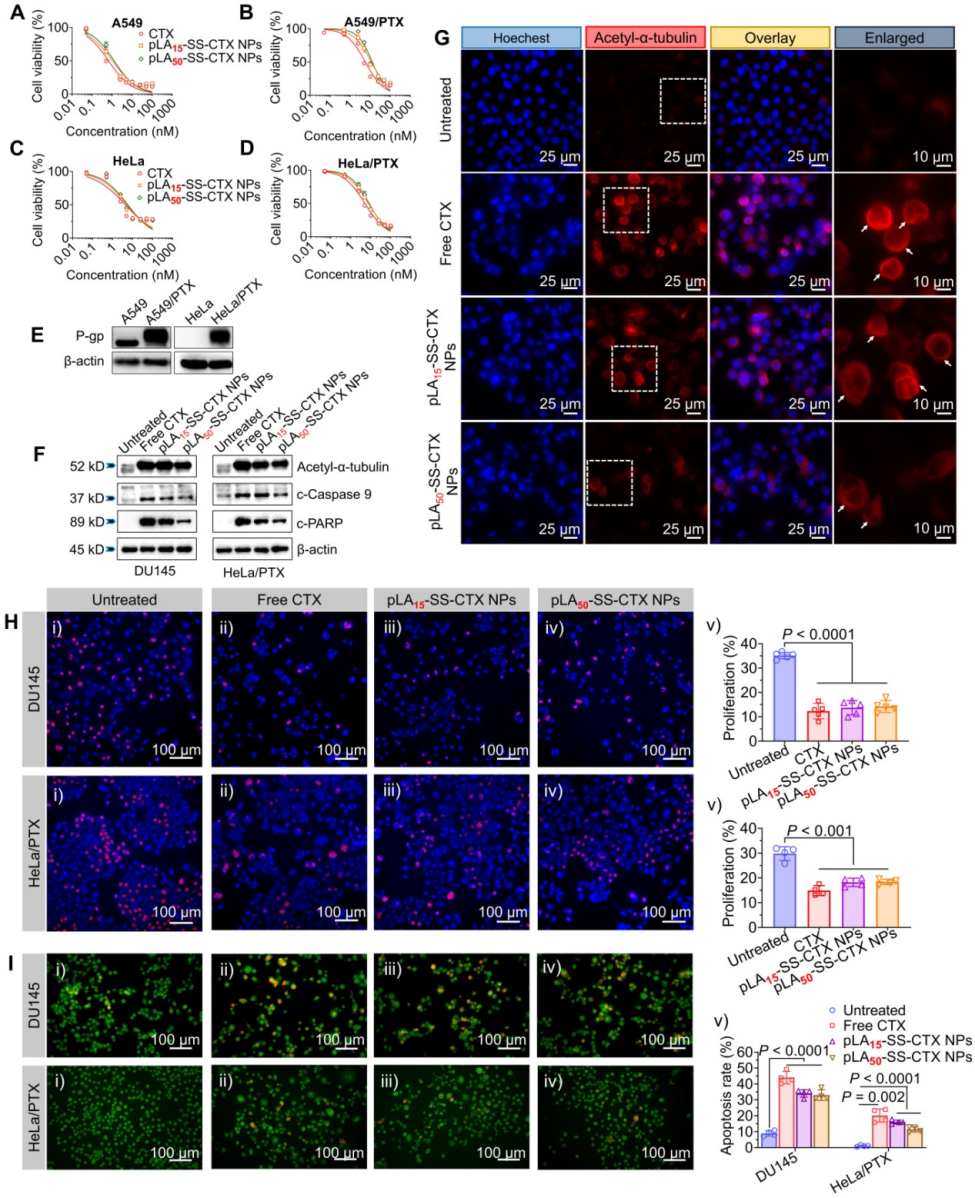
*In vitro* cytotoxicity of free cabazitaxel and pLA_n_-SS-CTX NPs (n = 15 or 50) against human cancer cell lines including **A**) A549, **B**) resistant A549/PTX , **C**) HeLa, **D**) resistant HeLa/PTX cells. **E**) P-gp levels in A549, A549/PTX, HeLa, and HeLa/PTX cells were assessed by western blot. **F**) Western blot analysis of apoptosis- and microtubule-associated proteins in DU145 and HeLa/PTX cells following the incubation with free cabazitaxel and pLA_n_-SS-CTX NPs (at a cabazitaxel-equivalent concentration, 10 nM for DU145 cells and 25 nM for HeLa/PTX cells).** G**) Representative fluorescence microscopy images suggest increased tubulin acetylation using the staining with acetyl-α-tubulin antibody. The cells were treated with free cabazitaxel and pLA_n_-SS-CTX NPs. **H**) Anti-proliferative effect of drugs (2 nM for DU145 cells and 6 nM for HeLa/PTX cells, cabazitaxel-equivalent concentration) determined by the Click-iT EdU assay. **I**) Acridine orange/ethidium bromide (AO/EB) assay for evaluation of cell apoptosis. Cells were treated with free cabazitaxel and pLA_n_-SS-CTX NPs for 48 h. The cell apoptotic ratio was defined as the ratio of apoptotic cells (brightly orange cells) to total cells (green cells). The data are presented as the means ± SD.

Next, the cytotoxic activities of pLA_n_-SS-CTX NPs were evaluated and compared with free cabazitaxel in a small panel of human cancer cell lines including A549, A549/PTX (paclitaxel-resistant cancer cell), HeLa, HeLa/PTX, and DU145 cells. As depicted in **Figure 4A-D** and **Figure S11**, after exposure to drugs for 72 h, the CCK8 assay was performed. Interestingly, the releasable pLA_n_-SS-CTX NPs exhibited comparably high cytotoxicity compared to free cabazitaxel in all tested cancer cell lines, as evidenced by the dose-response curves and IC_50_ values (**Table 2**). This cytotoxic behavior is quite distinct from our previously developed nonresponsive nanotherapies, which were approximately two orders of magnitude less effective than free cabazitaxel 27. These results suggested that once pLA_n_-SS-CTX NPs were internalized into cells, chemically unmodified drugs can be released spontaneously from the reservoir under the reducing cellular conditions and induce cancer cell apoptosis. However, the nonresponsive linker substantially impaired drug release from NPs in cells. The activities of the NPs were also tested in non-cancerous cells, including human umbilical vein endothelial cells (HUVEC) and murine macrophage cells (Raw 264.7). Activatable NPs showed significantly less cytotoxicity in these cells than free cabazitaxel, presumably due to low reducing agents in normal cells (**Table 2**). Therefore, exploiting the rational prodrug design ligated by a biologically activatable linkage, the selectivity towards cancer cells was accomplished in this nanoplatform.

The cytotoxic potency of pLA_n_-SS-CTX NPs was also assessed in two paclitaxel-resistant cancer cell lines (i.e., A549/PTX and HeLa/PTX cells), both of which were characterized by high levels of P-glycoprotein (P-gp, encoded by ABCB1 gene) using western blot analysis (**Figure 4E**). Moreover, both A549/PTX and HeLa/PTX cells were resistant to paclitaxel treatment, resulting in 209- and 22-fold increases in IC_50_ values compared with that of free cabazitaxel treatment, respectively. To our delight, in both resistant cells, free cabazitaxel and related nanotherapies retained high potency, without compromising their activity. These results are consistent with several previous results, indicating the low affinity of cabazitaxel toward P-gp and thereby highlighting their potential to treat taxane-resistant cancer 19.

The acetylation of α-tubulin K40 located in the lumen of microtubules was associated with microtubules polymerization. To explore whether pLA_n_-SS-CTX NPs stabilize microtubules by liberated cabazitaxel, we performed western blot analysis and immunofluorescence staining of acetyl-α-tubulin. The treatment with pLA_n_-SS-CTX NPs acetylated α-tubulin in tested DU145 and HeLa/PTX cells, which is in accordance with free cabazitaxel treatment (**Figure 4F**). Moreover, increased acetylation of α-tubulin was also observed in cabazitaxel- and pLA_n_-SS-CTX NP-treated cells under fluorescence microscopy observation (**Figure 4G**). The morphological changes after the NP treatment differed from untreated cells, suggesting that cabazitaxel can be activated in cells and potently inhibits the dissociation of microtubules. Eventually, released cabazitaxel triggered the classical caspase-dependent apoptosis as evidenced by the elevated expression of cleaved-caspase 9 (c-caspase 9) and cleaved-PARP (c-PARP) (**Figure 4F**) and resulted in cell death.

To evaluate the inhibitory effect of pLA_n_-SS-CTX NPs on cell proliferation, we conducted a Click-iT 5-ethynyl-2'-deoxyuridine (EdU) proliferation assay in DU145 and HeLa/PTX cells. Consistent with the cytotoxicity assay, pLA_n_-SS-CTX NPs induced comparable inhibition on the proliferation of cancer cells as free cabazitaxel (**Figure 4H**). The proliferation for drug treatments decreased from 35.1% ± 1.4% (untreated cells) to 12.4% ± 3.3% (cells treated with free cabazitaxel), 13.7% ± 2.9% (cells treated with pLA_15_-SS-CTX NPs), and 14.2% ± 2.4% (cells treated with pLA_50_-SS-CTX NPs) in DU145 cells and from 29.8% ± 2.8% (untreated cells) to 14.9% ± 2.0% (cells treated with free cabazitaxel), 18.2% ± 1.7% (cells treated with pLA_15_-SS-CTX NPs), and 18.4% ± 1.1% (cells treated with pLA_50_-SS-CTX NPs) in HeLa/PTX cells. Furthermore, as previously described, evident malformation was observed in the nuclei of the cells treated with free cabazitaxel and pLA_n_-SS-CTX NPs, which may be attributed to the inhibition on the disassembly of microtubules induced by cabazitaxel 27. The apoptosis-inducing ability of pLA_n_-SS-CTX NPs against DU145 and HeLa/PTX cells was evaluated by the acridine orange/ethidium bromide (AO/EB) staining method. AO can be incorporated into the DNA of all cells, emitting green fluorescence, while EB only binds to the DNA of apoptotic cells, exhibiting orange or red fluorescence. Cells treated with pLA_n_-SS-CTX NPs exhibited significant apoptosis (*p* < 0.001) compared to untreated cells, indicating that pLA_n_-SS-CTX NPs had potent apoptosis-inducing activity (**Figure 4I**).

Taken together, these data presented here revealed that pLA_n_-SS-CTX NPs had comparable *in vitro* cytotoxicity to that of free cabazitaxel. Covalent conjugation and nanoparticle encapsulation did not compromise the activity relative to its free drug form.

## Cellular uptake of NPs in cancer cells

**Figure 5:**
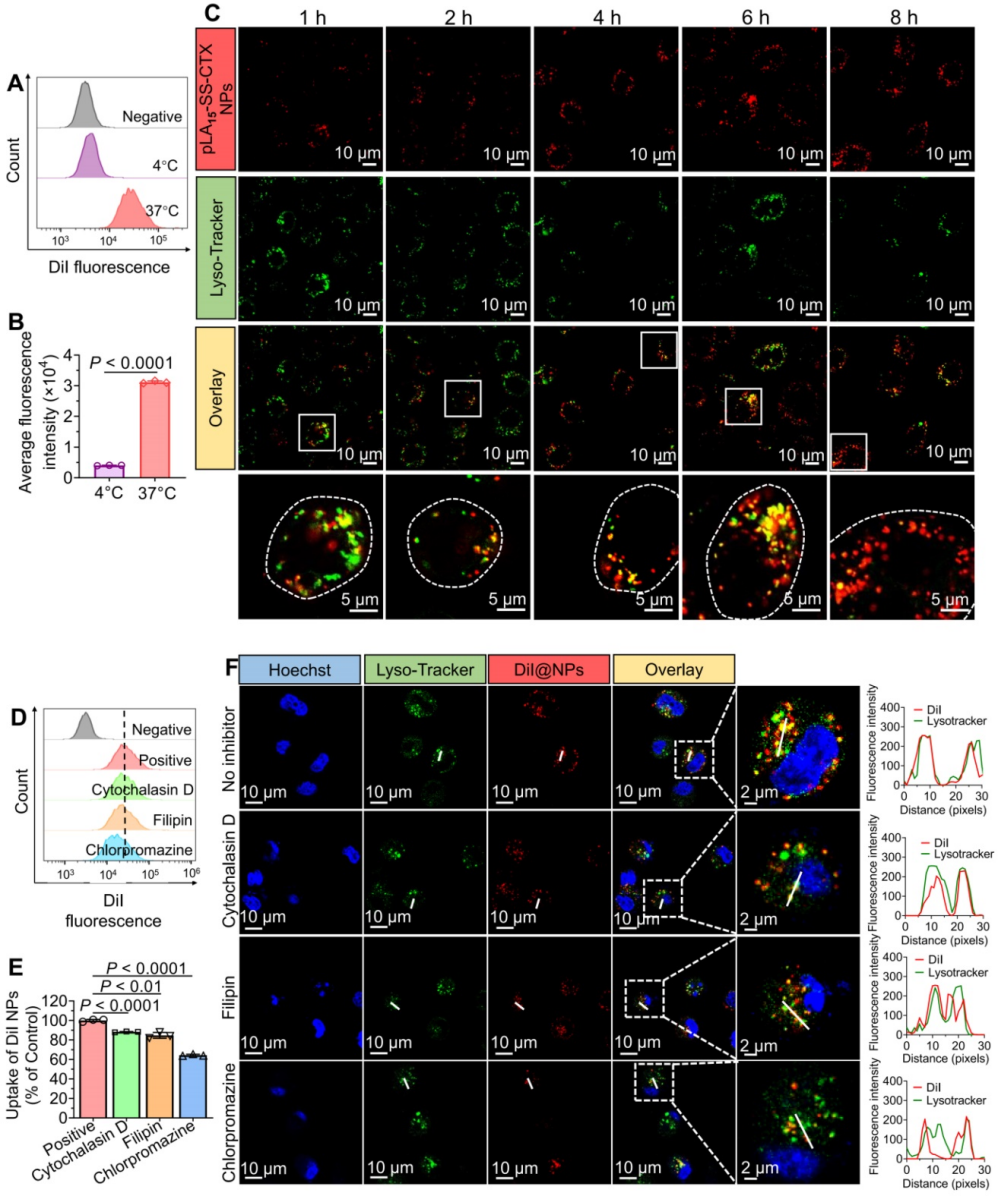
Cellular uptake and intracellular distribution of prodrug-loaded NPs in HeLa/PTX cells. (**A** and **B**) Flow cytometry analysis for uptake of DiI-labeled NPs after incubation at 37 °C or 4 °C. **C**) Time-lapse fluorescence images of HeLa/PTX cells to examine intracellular distribution of DiI-labeled NPs at 37 °C. Endo/lysosome compartments were stained with LysoTracker dye (green), and cell nuclei were stained with DAPI (blue). (**D** and **E**) Analysis of NP uptake in the presence or absence of inhibitors that target different endocytic pathways. **F**) Confocal laser scanning microscopy (CLSM)-based studies to visualize cytoplasmic delivery of pLA_n_-SS-CTX NPs. HeLa/PTX cells were pre-incubated with inhibitors and then treated with fluorescent NPs for 4 h. Representative CLSM images of HeLa/PTX cells pre-treated with chlorpromazine, an inhibitor of clathrin-dependent endocytosis, showed significantly reduced NP uptake. The data are presented as the means ± SD (n = 3).

To verify whether the active transport dominates the NP internalization, we incubated fluorescent dye DiI-labeled NPs with paclitaxel-resistant cells (HeLa/PTX cells) at 37 °C or 4 °C for 4 h. Compared with the active NP uptake at 37 °C, the uptake was significantly impaired when incubated at 4 °C, which was in accordance with the energy-dependent endocytosis mechanism (**Figure 5A-B**). To further explore the uptake pathway and intracellular distribution of the NPs, cells were incubated with DiI-labeled pLA_15_-SS-CTX NPs (red fluorescence) at 37 °C. Concurrently, cells were stained with LysoTracker Green to mark endo/lysosomal compartments. The time-lapse fluorescence imaging showed that the NPs were trafficked to these compartments, as highlighted by their good initial colocalization with LysoTracker (**Figure 5C**). Subsequently, the red signals derived from the NPs increased over time and did not colocalize with LysoTracker green at 8 h, indicating that the NPs might escape from endo-lysosomes to release drug payloads in the cytoplasm.

We next investigated the specific endocytic pathway of pLA_n_-SS-CTX NP uptake by incubation with various inhibitors. The cells were pre-treated with distinct inhibitors for 30 min, and then DiI-labeled NPs were incubated for another 4 h. As shown in **Figure 5D-E**, chlorpromazine, an inhibitor of clathrin-dependent endocytosis, caused a reduction in NP uptake to 64.4%±1.0%. Intriguingly, cytochalasin D and filipin III (inhibitors of pinocytosis and caveolin-mediated endocytosis, respectively) also significantly reduced the internalization of pLA_n_-SS-CTX NPs. Therefore, we concluded that clathrin-mediated endocytosis was the major pathway for pLA_n_-SS-CTX NPs. However, pinocytosis and caveolin-mediated endocytosis were also involved in the cellular uptake of this therapeutic NP. Subsequently, we exploited confocal laser scanning microscopy (CLSM) to determine the cellular uptake of DiI-labeled NPs. As expected, cellular uptake of pLA_n_-SS-CTX NPs was markedly blocked by chlorpromazine (**Figure 5F**), consistent with the results derived from flow cytometry analysis. Intriguingly, pre-incubation with chlorpromazine resulted in reduced colocalization of NPs with lysosomes, presumably due to impaired endocytosis.

## Tumor accumulation of nanotherapies

**Figure 6:**
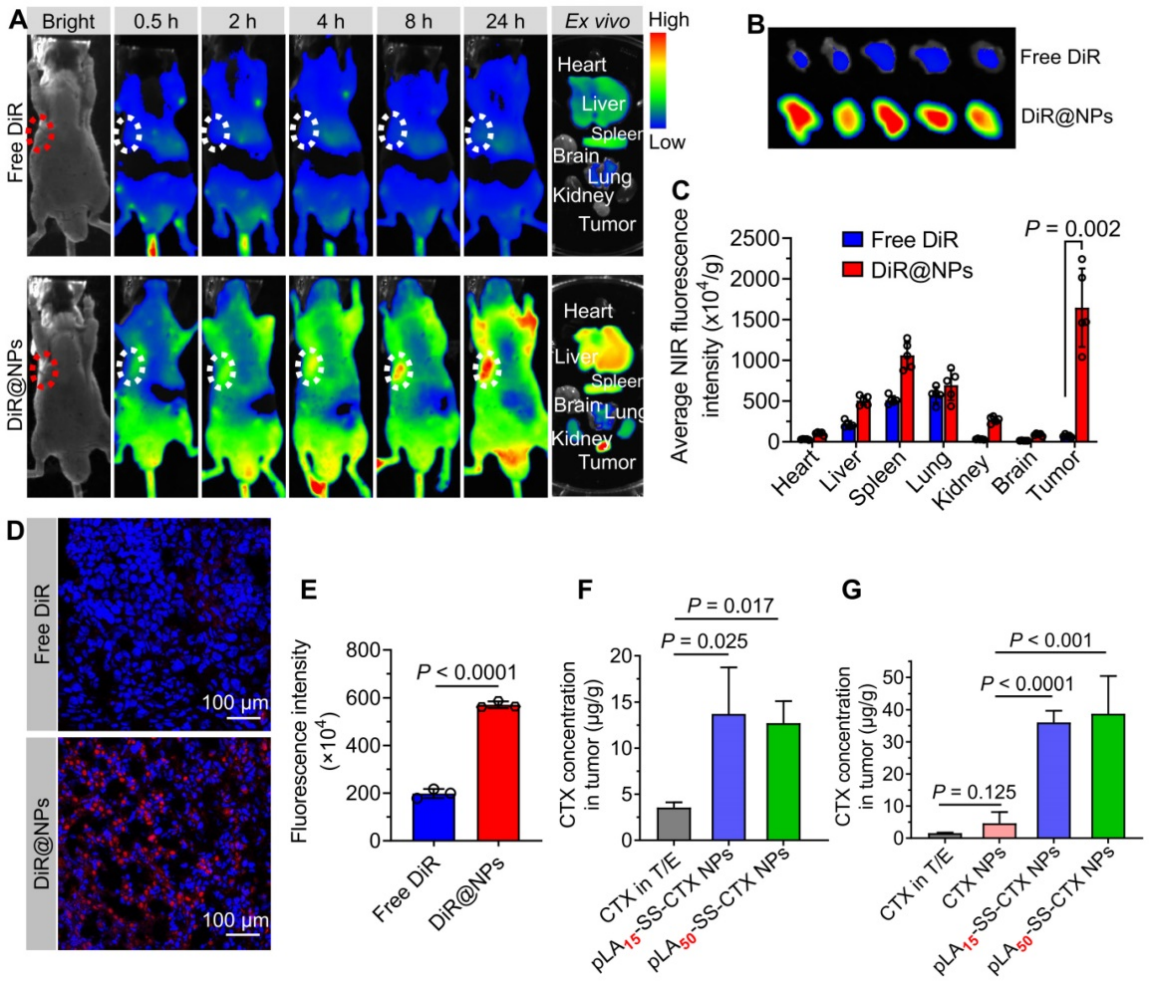
Evaluation of the tumor-targeting capability of NPs in a HeLa/PTX xenograft-bearing mouse model. **A**) *In vivo* fluorescence imaging of mice treated with free DiR and fluorescent DiR-labeled NPs. Excised tumors and major tissues (e.g., heart, liver, spleen, lung, kidney, and brain) were also photographed at 24 h post-injection. Dotted circles indicate the tumor regions. **B**) Images of tumors from free DiR and NP treated mice. **C**) Quantitative analysis of average NIR fluorescence intensity in tumors and other tissues (n = 5). **D** and **E**) Fluorescence images and intensity of the tumor sections visualized by CLSM. Drug concentrations in mouse models of the human prostate DU145 (**F**) and paclitaxel-resistant cervical A549/PTX tumor xenografts (**G**) after dosing of free cabazitaxel, free cabazitaxel-loaded NPs and pLA_n_-SS-CTX-NPs. The data are presented as the means ± SD.

A preclinical model of HeLa/PTX xenografts in Balb/c nude mice was established to evaluate the tumor-targeting capability of pLA_n_-SS-CTX-NPs. The near-infrared (NIR) fluorescence probe DiR was utilized to label the NPs for tracking the *in vivo* distribution. DiR dissolved in polysorbate 80/ethanol (1:1, v/v) was included as a reference. Following a single injection *via* the tail vein, whole-body fluorescence imaging was performed with an *in vivo* imaging system at predetermined time points. As shown in **Figure 6A**, free DiR-treated mice exhibited a much lower fluorescence signal than NP-treated mice, suggesting the rapid clearance of free DiR in animals. Notably, NP treatment resulted in substantial drug delivery to the tumor sites, whereas negligible tumor accumulation was confirmed in the mice treated with free DiR (**Figure 6B**). To further elucidate the biodistribution of NPs, the mice were sacrificed at 24 h post-administration. *Ex vivo* imaging of major organs and tumors revealed that free DiR mainly accumulated in the liver, spleen, and lung, due to rapid metabolism *in vivo* (**Figure 6A** and **C**). Interestingly, nanodelivery significantly increased fluorescent signals in tumors. In NP-treated mice, the average intensities in tumors were 3.2-fold higher than those in the liver, 2.4-fold higher than those in the lung, and 1.6-fold higher than those in the spleen, suggesting that pLA_n_-SS-CTX-NPs altered the distribution and favored drug accumulation in tumors.

We further investigated the tumor distribution of the NPs using CLSM observation. Compared to the weak fluorescence observed in free DiR-treated mouse tumors, NP-treated tumor sections presented substantially stronger and homogeneous signals (**Figure 6D-E**).

To further confirm whether NP delivery can increase the drug concentration at tumor sites, pLA_n_-SS-CTX-NPs were intravenously injected into tumor-bearing mice at a dose of 25 mg/kg (cabazitaxel-equivalent dose). Free cabazitaxel in the Jevtana^®^-mimicking formulation (polysorbate 80/ethanol, 1:1, v/v) and free cabazitaxel-loaded PEG_5k_-*b*-PLA_8k_ polymeric NPs (termed CTX NPs) were included as comparisons. At 24 h post-injection, the tumor tissues were excised, and drug concentrations were determined by HPLC analysis. Indeed, significant higher levels of cabazitaxel drugs accumulated in the tumors of pLA_n_-SS-CTX-NPs-treated mice relative to that of free cabazitaxel or CTX NP-treated mice (**Figure 6F-G**). Given the augmented compatibility of the prodrugs with the encapsulation matrices, pLA_n_-SS-CTX NPs showed favorable stability and low premature release of toxic drugs in the bloodstream, which could facilitate intratumoral accumulation *via* the enhanced permeability and retention effect.

## Antitumor efficacy in a paclitaxel-sensitive prostate model

**Figure 7:**
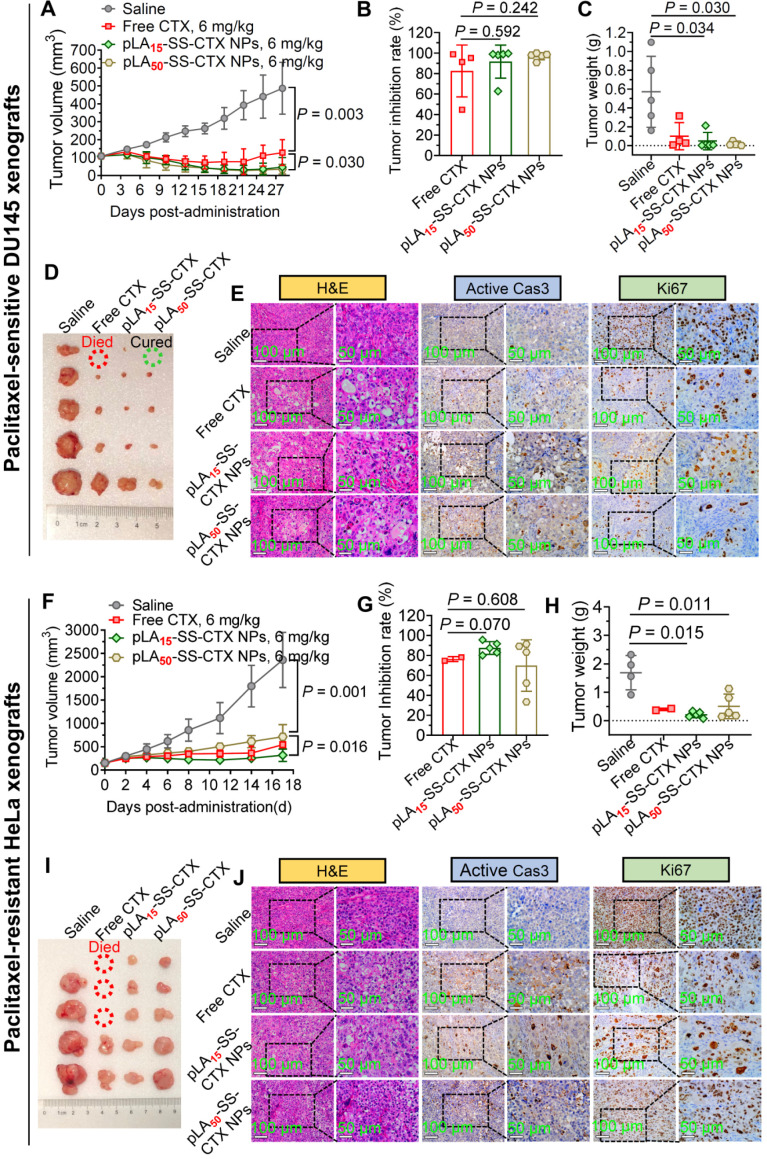
Antitumor activity of free cabazitaxel and pLA_n_-SS-CTX NPs against human prostate DU145 and paclitaxel-resistant cervical HeLa/PTX tumor xenografts. Mice were intravenously injected with free cabazitaxel and pLA_n_-SS-CTX NPs at a cabazitaxel-equivalent dose of 6 mg/kg for three successive times. **A** and **F**) Tumor growth curves upon drug treatment. **B** and **G**) Tumor inhibition rates of free cabazitaxel and NPs in comparison to the saline treatment. **C** and **H**) Tumor weights in each group. **D** and **I**) Photographs of the tumors excised from each treatment group at the endpoint of the study. **E** and **J**) H&E, active (cleaved) caspase-3, and Ki67 staining of the tumor sections. Scale bars: 100 µm. The data are presented as the means ± SD.

Encouraged by excellent cytotoxicity and preferential tumor accumulation endowed by NP delivery, we evaluated the therapeutic efficacy in preclinical models of human tumor xenografts. Currently, cabazitaxel formulation (Jevtana^®^) has been approved for the treatment of prostate cancer in the clinic 29. We therefore first assessed the activity of the NPs in a human prostate DU145 cancer model. Tumor xenografts were established in Balb/c nude mice, and therapy was initiated by intravenously administering drugs *via* the tail vein when the tumors reached ~100 mm^3^ in volume. After dosing free cabazitaxel in a pharmaceutical Jevtana^®^-mimicking formulation (polysorbate 80/ethanol, 1:1, v/v) at 6 mg/kg, tumor growth was suppressed, but rebounded immediately after cessation of treatment. Injection of the NPs at the same dose resulted in tumor shrinkage in this model (**Figure 7A**, **C-D**). The efficacy of NP treatments lasted over the observation period. The average tumor inhibition rates for free cabazitaxel, pLA_15_-SS-CTX NPs, and pLA_50_-SS-CTX NPs were 83%, 92%, and 97%, respectively, at the endpoint of the study (**Figure 7B**). However, there was no statistical difference between the two NP treatment groups.

Hematoxylin and eosin (H&E) staining of the tumor tissues was shown in **Figure 7E**. Obviously, the tumors were dramatically damaged after drug treatment. For instance, vacuolization and karyopyknosis were extensively observed in the tumor sections, indicating intratumoral apoptosis induced by cytotoxic cabazitaxel. In contrast, in saline-treated mouse tumors, the cells were packed tightly with full chromatin and nuclei. Caspase-3 can be activated by the cleavage of procaspase-3, which serves as a characteristic marker of apoptosis 30. Hence, elevated active caspase-3 suggested that NP treatment led to efficient tumor cell apoptosis. Ki67, a specific marker of cell proliferation, was also stained to assess the antitumor activity of the NPs 31. Consistently, the decreased levels of Ki67 in tumors verified that NP treatment efficiently inhibited the proliferation of tumor cells, which produced superior tumor suppression.

## Superior *in vivo* activity of prodrug-loaded therapy against paclitaxel-resistant models

Cabazitaxel has low affinity towards P-gp transporters; thus, this drug is capable of overcoming drug resistance induced by paclitaxel or docetaxel. We therefore assessed the activity of these therapeutics in a paclitaxel-resistant HeLa/PTX cervical tumor model. Cabazitaxel in the Jevtana^®^-mimicking formulation suppressed tumor growth but caused significant mouse death (**Figure 7I**). Satisfyingly, the efficacy of the nanotherapies was superior to cabazitaxel administered in solution. Mice treated with pLA_15_-SS-CTX NPs had slower tumor outgrowth than mice treated with pLA_50_-SS-CTX NPs (*p* < 0.05) in this resistant HeLa/PTX model (**Figure 7F-H**). At the endpoint of the study, the average tumor volume of the pLA_15_-SS-CTX NP-treated group was 319 mm^3^, which was half of the tumor volume in mice receiving pLA_50_-SS-CTX NPs. On the basis of the therapeutic responses observed in two xenograft tumor models, we showed that the combination of the prodrug approach with nanotherapies was valuable for drug delivery, particularly for pLA_15_-SS-CTX NPs, showing higher safety compared to free cabazitaxel, leading to more efficient tumor inhibition than pLA_50_-SS-CTX NPs. In addition, histological analysis of tumors after treatments was performed to further verify the antitumor activity. A similar trend observed in the paclitaxel-sensitive DU145 prostate model was also confirmed in this model. Assays of H&E, active caspase-3, and Ki67 revealed that intensive intratumoral apoptosis was caused by treatments with cabazitaxel and nanotherapies (**Figure 7J**).

To further examine the strategy that balances NP stability and specific drug activation, we compared the* in vivo* potency of pLA_15_-SS-CTX NPs with free cabazitaxel-loaded PEG_5k_-*b*-PLA_8k_ NPs in two taxane-resistant models of human cancer. In HeLa/PTX and A549/PTX tumor xenograft-bearing models, pLA_15_-SS-CTX NPs were more effective to suppress tumor growth than CTX NPs (**Figure S12-13**). Noticeably, the prodrug nanotherapy was less toxic than CTX NPs in animals, as evidenced by stable body weight. Histological analyses such as H&E, active caspase-3, and Ki67 staining also confirmed the effectiveness of pLA_15_-SS-CTX NPs (**Figure S14**). Overall, these *in vivo* experiments provided convincing evidence that activatable prodrug NPs could be a promising platform for further efficacy testing in other aggressive tumor models.

## Efficacy of the prodrug nanotherapy for the treatment of recurrent tumors

To demonstrate the efficacy of the nanotherapies, we further established a recurrent tumor model pretreated with cabazitaxel (**Figure S15**). Briefly, HeLa/PTX cervical tumors in mice were pretreated with free cabazitaxel (6 mg/kg) every three days for six times, and subsequently, the tumors were re-transplanted into Balb/c nude mice. The successive intravenous injection of free cabazitaxel resulted in elevated expression of class III β-tubulin (TUBB3, **Figure S15A**), which is associated with alternations in microtubule dynamicity and cabazitaxel resistance 32. In this recurrent tumor model, treatment of pLA_15_-SS-CTX NPs was able to suppress tumor burden significantly, lasting for two weeks (**Figure S15B**). However, CTX NPs failed to effectively inhibit tumor growth, and the tumors recurred on day 6 after the last administration. Together, this result substantiates the potential utility of our prodrug nanoparticle approach to treat recurrent tumors with partial drug resistance.

## Safety assessment of nanotherapies

**Figure 8:**
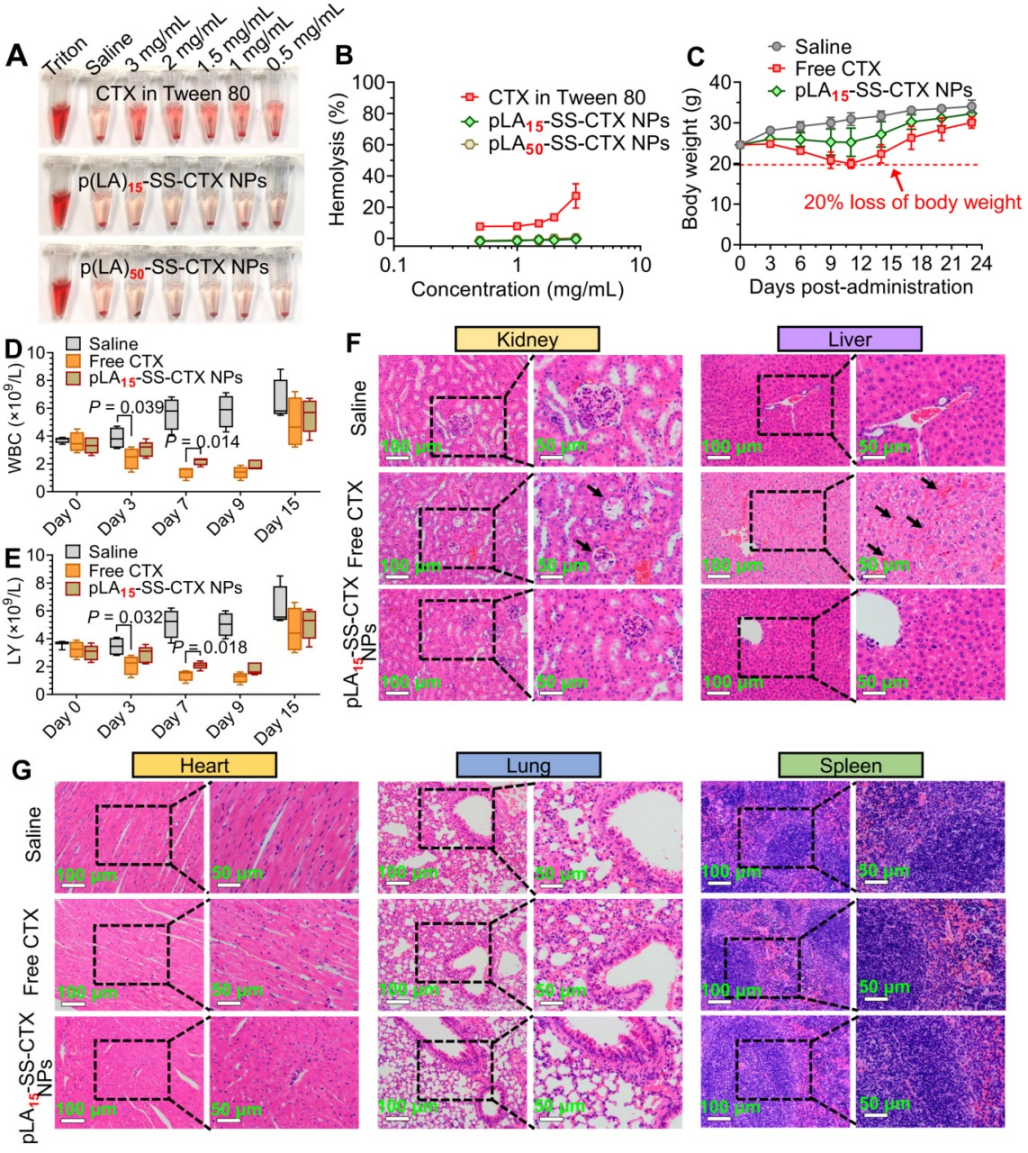
*In vivo* safety assessment of the NPs compared with free cabazitaxel. **A** and **B**) Hemolysis of the clinical formulation of cabazitaxel, pLA_15_-SS-CTX-NPs and pLA_50_-SS-CTX-NPs at a series of concentrations. Saline was included as a negative control, while 1% Triton X-100 was included as a positive control. Changes in body weights **C**), white blood cells (WBCs) **D**) and lymphocytes (LYs) **E**) following drug administration. The data are presented as the means ± SD. **F** and** G**) H&E staining of major organs from each group.

The biocompatibility of pLA_n_-SS-CTX NPs for intravenous administration was first evaluated by the incubation of drug formulations with red blood cells (RBCs). A dramatic hemolysis of RBCs was observed after incubation with cabazitaxel in the Jevtana^®^-mimicking formulation. At a drug concentration of 3 mg/mL, this formulation presented a significantly high ratio of hemolysis, reaching 27.3% (**Figure 8A-B**). In contrast, both nanotherapies showed negligible hemolysis even when the drug concentration was increased to 3 mg/mL. Thus, we concluded that the nanoplatforms were sufficiently applicable for intravenous dosing.

Owing to the superior activity observed in HeLa/PTX xenografts, we assessed the toxicity of pLA_15_-SS-CTX NPs in healthy ICR mice. The mice administered with free cabazitaxel exhibited a substantial reduction in body weight (~20%), whereas the body weight remained stable in pLA_15_-SS-CTX NP-treated mice (**Figure 8C**). Neutropenia is considered a major dose-limiting side effect for cabazitaxel 33. Therefore, we verified whether the NP approach could mitigate neutropenia-associated side effects by testing the counts of white blood cells (WBCs) and lymphocytes (LYs). **Figure 8D-E** showed that free cabazitaxel induced reductions in the counts of WBCs and LYs after single drug injection. Satisfyingly, no statistical reduction was caused by the NPs at the same dose. Following three injections, NP treatment exhibited slight myelosuppression but rebounded to the normal range after cessation of treatment.

Finally, histological analyses of major organs (e.g., heart, liver, spleen, lung, and kidney) were performed to verify the damage induced by dosing the drugs. Unfortunately, damage to the liver and kidney was observed in cabazitaxel-administered mice. In addition, severe hepatorrhagia, renal tubular epithelial cells, and glomerulus necrosis were also found in cabazitaxel-treated mice (**Figure 8F**). In contrast, the livers and kidneys of nanotherapy-treated mice presented similar histological patterns to those of saline-treated mice, suggesting a potentially negligible toxicity of NP treatment (**Figure 8G**).

## Discussion and Conclusion

On-demand smart drug delivery in the context of anticancer therapy is a longstanding goal with the capacities for tumor-enriched accumulation and triggered drug release 34. With this design rationale in mind, we selected cabazitaxel for testing the validity of our “balancing nanoparticle stability and specific drug activation” strategy. Cabazitaxel, a mitotic inhibitor, is a potent chemotherapeutic drug candidate for cancer treatment. As a new generation of taxane drugs, cabazitaxel holds great therapeutic potential to overcome drug resistance due to its low affinity for multidrug resistance proteins and evasion of drug efflux. However, cabazitaxel exhibited high dose-limiting toxicities in clinical trials 9. The maximum tolerated dose (MTD) of cabazitaxel has been established as 25 mg/m^2^, being significantly lower than those of paclitaxel and docetaxel (175 mg/m^2^ for paclitaxel, 60-100 mg/m^2^ for docetaxel, 1-hour infusion every 3 weeks) 35, 36. Consequently, cabazitaxel therapy has only limited clinical success, with a single FDA approval to date for the treatment of mCRPC after docetaxel therapy 37. Currently, free cabazitaxel is formulated in polysorbate 80 and diluted with 13% ethanol aqueous solution, under the trademark of Jevtana, for intravenous injection 38. Therefore, a sensitivity reaction attributable to polysorbate 80 is occasionally observed in Jevtana^®^-administered patients. Nonetheless, alternative strategies without the use of surfactants are desperately desired to achieve optimal therapeutic outcomes and good safety profiles 39-41.

Recently, an increasing number of polymeric nanoscopic platforms have been developed for the delivery of active compounds 42-49. In many nanoparticle scaffolds, therapeutics are commonly encapsulated in matrices *via* noncovalent interactions, which may result in suboptimal pharmacokinetic properties and insufficient drug concentrations in diseased sites. For example, Genexol-PM, which is a PEG-*b*-PLA-comprising delivery vehicle encapsulating pure paclitaxel, showed low systemic toxicity, but higher doses did not improve the overall survival in patients 50, 51. These clinical data may implicate the unsuccessful vectorization of taxane agents, which motivates us to develop alternative strategies for efficient anticancer drug delivery. Several groups, including us, have recently exploited hydrophobic polylactide segments to molecularly edit drugs to augment the affinity between drug compounds and nanocarriers 27, 52, 53. A prodrug strategy was included to conjugate the active drugs to the polymers for subsequent NP preparation. With the enhanced compatibility and miscibility between the prodrugs and the encapsulation matrices, the nanotherapies had prolonged circulation in the bloodstream, which could contribute the increased intratumoral drug accumulation. Unfortunately, in some cases, the low release efficiency of cytotoxic cargoes from the NPs compromised the anticancer activity and further reduced the drug potency. Here, we speculated that tumor environment-stimulated drug release could be taken into consideration for improving the efficacy of covalently conjugated drugs. The disulfide bond is highly susceptible to reduced GSH that is abundant in cancer cells and can be properly used for drug activation, while this type of conjugate remains sufficiently stable in the blood circulation 54, 55. Intrigued by this idea, we devised reduction-activatable linkage into the polyprodrug constructs. To facilitate the synthesis of prodrugs and achieve desired drug loading and prolonged circulation, the polylactide segment was selected to molecularly edit drugs. The resultant pLA_n_-SS-CTX conjugates showed rapid release of active cabazitaxel in response to GSH compared with the cabazitaxel prodrugs ligated by the ester bond linkage.

PTX-resistant A549 and HeLa cells were established to test the efficacy of these nanotherapies. Compared with the drug-sensitive tumor cells, both A549/PTX and HeLa/PTX cell lines were highly resistant to paclitaxel treatment, as evidenced by the cell viability assay. Moreover, the resistance yielded by the overexpression of P-gp was identified by western blot analysis (**Figure 4E**). Using these cell lines, we tested the efficacy of solution-based free drug and the prodrug NPs. Unlike paclitaxel treatment, cabazitaxel-based therapeutics remained effective in inducing cell apoptosis. Covalent conjugation and NP encapsulation did not compromise the *in vitro* cytotoxic activity relative to its free drug form. The excellent cytotoxicity of the prodrug NPs was consistent with the drug release profiles, highlighting our design rationale of integrating a bioactivatable linker for rapid intracellular drug activation.

Compared with the cabazitaxel agent in the Jevtana^®^-mimicking formulation and the PEG_5k_-*b*-PLA_8k_ NPs, the prodrug NPs exhibited higher drug accumulation at tumor sites. For example, when the pLA_15_-SS-CTX and pLA_50_-SS-CTX NPs were intravenously administered, drug concentrations were 3.9- and 3.6-fold, respectively, higher than that of free cabazitaxel in DU145 tumors. Furthermore, in A549/PTX tumors, the pLA_15_-SS-CTX and pLA_50_-SS-CTX NPs exhibited 23.0- and 24.7-fold, respectively, higher than that of free cabazitaxel and 7.7- and 8.3-fold, respectively, higher than that of CTX NPs. Intrigued by favorable tumor targeting, we evaluated the potential of the prodrug NPs against three independent xenograft tumor models, including paclitaxel-sensitive DU145 xenograft and paclitaxel-resistant HeLa/PTX and A549/PTX xenografts. Administrations of the prodrug NPs resulted in superior antitumor activity and improved safety profiles relative to free cabazitaxel and free cabazitaxel-loaded NPs. Finally, pLA_15_-SS-CTX NPs substantially alleviated drug toxicity, as supported by the stable mouse body weights and negligible histological damage in major organs.

In conclusion, the attachment of toxic cabazitaxel to polylactides *via* a self-immolative linkage and subsequent nanoparticle formulation generated injectable nanosystems for safe and effective drug delivery. By varying the chain length of the polylactide modifier, the balance between stability during circulation and drug activation kinetics in tumor cells was tailored for efficacy optimization. Furthermore, by rendering the cabazitaxel agent inactive temporarily through a bioactivatable disulfide linker, the drug could be tolerated at higher doses. Collectively, the approach described here improved efficacy and reduced drug toxicity, which could be a new generalizable strategy for the formulation of other hydrophobic anticancer agents.

## Supplementary Material

Supplementary materials including figures.

## Figures and Tables

**Table 1:** Characterization of pLA_n_-SS-CTX-loaded nanoparticles.

	Size (nm)	PDI	Zeta potential (mV)	EE (%)*^a^*	DL (%)*^b^*
pLA_15_-SS-CTX NPs	79.9±1.9	0.164±0.002	-4.47±0.45	95.10±1.00	4.33±0.05
pLA_50_-SS-CTX NPs	93.8±4.8	0.135±0.028	-4.34±0.74	96.91±1.07	3.91±0.04

*a*, Drug encapsulation efficiency; *b*, Drug loading.

**Table 2:** IC_50_ values extrapolated from dose-response curves shown in **Figure 4A**-**D and Figure S11**. Cells were treated with free cabazitaxel or pLA_n_-SS-CTX NPs (n = 15 or 50), and the cell viability was determined by the CCK8 assay. The data are presented as the means ± SD.

Drug formulations	IC_50_ (nM)							
	Cancer cells				Normal cells			
	DU145	A549	A549/PTX	HeLa		HeLa/PTX	HUVEC	Raw 264.7
Free PTX	4.15±0.29	3.36±0.35	701.20±61.85	5.85±0.98		127.30±11.00		
Free cabazitaxel	1.88±0.12	0.66±0.10	5.70±0.51	4.34±0.74		5.11±0.35	0.62±0.06	3.60±0.30
pLA_15_-SS-CTX NPs (Fold increased)^a^	2.38±0.12 (1.27)	1.04±0.15 (1.58)	11.42±0.90 (2.00)	5.19±0.81 (1.20)		7.65±0.34 (1.50)	2.95±1.22 (4.76)	14.25±0.77 (3.96)
pLA_50_-SS-CTX NPs (Fold increased)^b^	2.44±0.11 (1.30)	1.25±0.18 (1.89)	12.68±0.99 (2.22)	5.77±0.86 (1.33)		8.32±0.40 (1.63)	4.14±1.37 (6.68)	21.27±1.12 (5.91)
								

^a^ Fold increased: IC_50_ (pLA_15_-SS-CTX NPs)/ IC_50_ (free cabazitaxel); ^b^ Fold increased: IC_50_ (pLA_50_-SS-CTX NPs)/ IC_50_ (free cabazitaxel).
