# Supplementary material for: Balancing the stability and drug activation in adaptive nanoparticles potentiates chemotherapy in multidrug-resistant cancer
Source: Theranostics. 2021 Feb 19;11(9):4137–54. doi: 10.7150/thno.54066 (PMC7977460; doi:10.7150/thno.54066)
Supplement: Supplementary file 1 — Supplementary materials including figures. [file thnov11p4137s1.pdf]

Supporting Information

Balancing the stability and drug activation in adaptive nanoparticles potentiates chemotherapy in multidrug-resistant cancer

Jianqin Wan,[a] Lingling Huang,[b] Jiangting Cheng,[a] Qi Huangfu,[a] Jiahui Jin,[c] and Hangxiang Wang[a,]*

[a] The First Affiliated Hospital, Zhejiang University School of Medicine; NHC Key Laboratory of Combined Multi-Organ Transplantation; Key Laboratory of Organ Transplantation, Research Center for Diagnosis and Treatment of Hepatobiliary Diseases, Zhejiang Province, Hangzhou, P. R. China.

[b] Institute of Pharmaceutics, College of Pharmaceutical Sciences, Zhejiang University, Hangzhou, Zhejiang 310058, P. R. China.

[c] Xingzhi College, Zhejiang Normal University, Jinhua, Zhejiang 321004, P. R. China.

Corresponding Authors: *Hangxiang Wang, The First Affiliated Hospital, Zhejiang University School of Medicine; 79, Qingchun Road, Hangzhou 310003, China. Phone: +86-571-88208157; E-mail: wanghx@zju.edu.cn*

Materials and methods

Cabazitaxel (CTX) was purchased from Knowshine Pharmachemicals Inc. (Shanghai, China). Carbocyanine (DiR) was purchased from Thermo Fisher Scientific (USA). mPEG$_{5k}$-*b*-PLA$_{8k}$ was obtained from Daigang Biomaterial Co., Ltd (Jinan, China). All reagents were obtained from commercial suppliers (Sigma-Aldrich, Tokyo Chemical Industry (TCI), or J&K Chemical) and used without further purification.

Analytical RP-HPLC was carried out on a Hitachi Chromaster 5000 system equipped with a YMC-Pack C8 ODS reverse-phase column (5 μm, 250 × 4.6 mm, YMC Co., Ltd., Kyoto, Japan) at a flow rate of 1.0 mL/min. A gradient of 30%-100% acetonitrile in water containing 0.1% TFA within 20 min was employed as the mobile phase. UV detection was performed at 220 nm. TEM images of pLA$_n$-SS-CTX NPs were obtained using TECNAL 10 (Philips) at an acceleration voltage of 80 kV. The hydrodynamic diameters (D_H) were analyzed on a Malvern Nano-ZS90 instrument (Malvern Instruments, Malvern, UK) at 25° C.

General methods for organic synthesis

All reactions were performed in a dry atmosphere. Thin layer chromatography (TLC) was performed on silica gel 60 F_{254} pre-coated aluminium sheets (Merck) and visualized by fluorescence quenching. Chromatographic purification was accomplished using flash column chromatography on silica gel (neutral, Qingdao Haiyang Chemical Co., Ltd). ^{1}H NMR spectra were recorded in $CDCl_3$ on a Bruker 400 (400 MHz) spectrometer and calibrated to the residual solvent peak or tetramethylsilane (= 0 ppm). Multiplicities are abbreviated as follows: s = singlet, d = doublet, t = triplet, m = multiplet, br = broad.

Synthesis of hydroxyl-terminated polylactide (pLA$_{15}$)

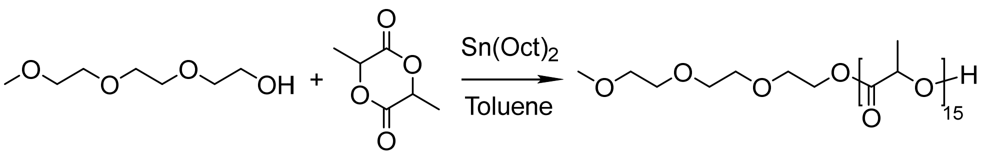

Hydroxyl-terminated polylactide (pLA$_{15}$) was synthesized by ring-opening polymerization. Briefly, $_{D,L}$-lactide (91.57 mmol, 13.19 g) and

triethylene glycol monomethyl ether (11.02 mmol, 1.81 g) dissolved in 50 mL toluene were added into a round-bottom flask, followed by an addition of stannous octoate (Sn(Oct)$_2$). After stirring for 12 h at 140° C, the solvent was removed by evaporation under vacuum. Subsequently, the residue was dissolved in dichloromethane (DCM) and then precipitated in excess cold diethyl ether and filtered. The molecular weight of pLA$_{15}$ was determined by ^{1}H NMR.

^{1}H NMR (400 MHz, CDCl$_3$): δ 5.26-5.13 (m, 15H), 4.38-4.25 (m, 2H), 3.71-3.68 (m, 2H), 3.64-3.63 (m, 6H), 3.56-3.53 (m, 2H), 3.38 (s, 3H), 1.59-1.52 (m, 45H).

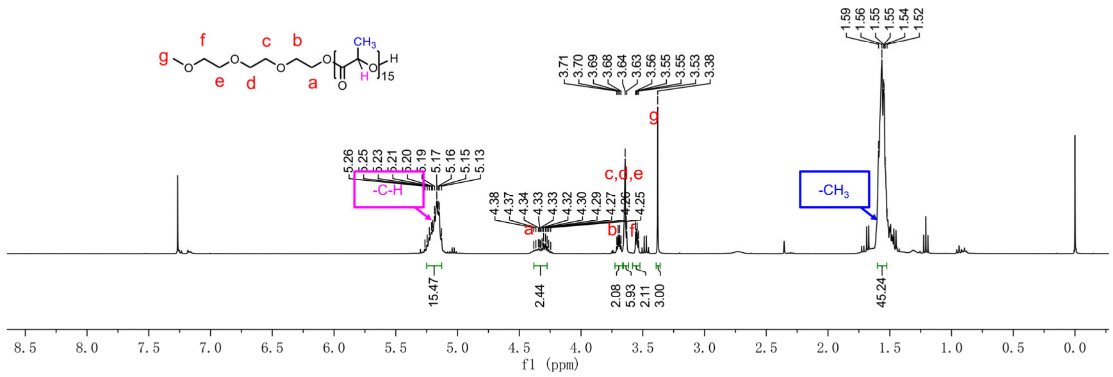

Figure S1. ^{1}H NMR spectrum of hydroxyl-terminated polylactide (pLA$_{15}$) in CDCl$_3$.

Synthesis of hydroxyl-terminated polylactide (pLA$_{50}$)

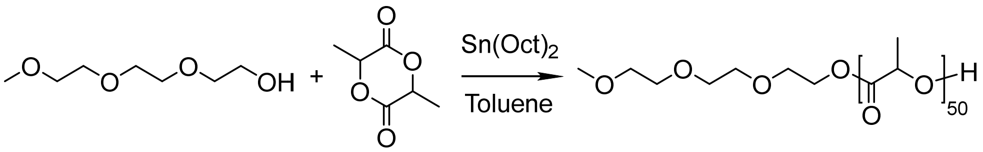

Hydroxyl-terminated polylactide (pLA$_{50}$) was also synthesized by ring-opening polymerization. Briefly, $_{\text{D,L}}$-lactide (120.45 mmol, 17.35 g) and triethylene glycol monomethyl ether (3.96 mmol, 0.65 g) dissolved in 50 mL toluene were added into a round-bottom flask, followed by an addition of stannous octoate (Sn(Oct)$_2$). After stirring for 12 h at 140° C, the solvent was removed by evaporation under vacuum. Subsequently, the residue was dissolved in DCM and then precipitated in excess cold diethyl ether and filtered. The molecular weight of pLA$_{50}$ was determined by ^{1}H NMR.

^{1}H NMR (400 MHz, $CDCl_3$): δ 5.25-5.13 (m, 50H), 4.38-4.26 (m, 2H), 3.70-3.68 (t, *J* =8, 2H), 3.65-3.63 (m, 6H), 3.56-3.53 (m, 2H), 3.38 (s, 3H), 1.59-1.55 (m, 150H).

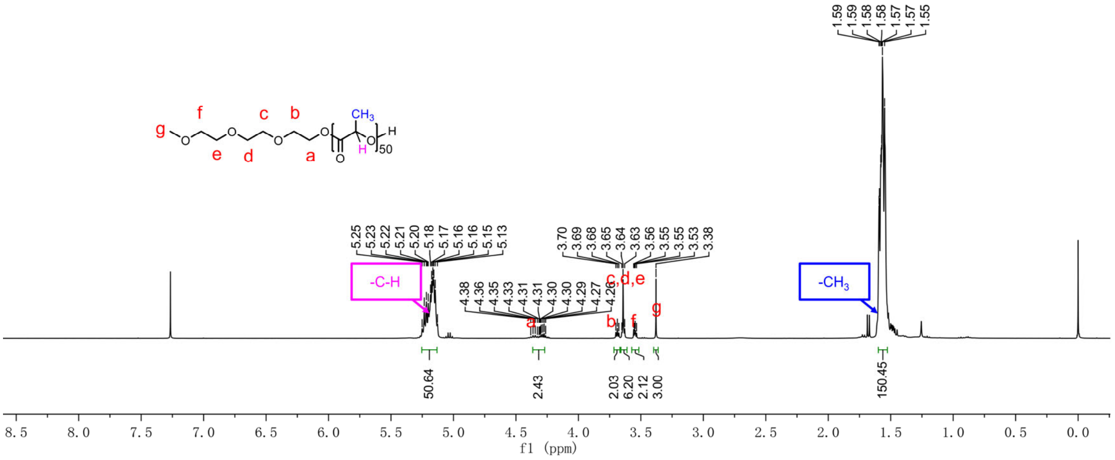

Figure S2. ^{1}H NMR spectrum of hydroxyl-terminated polylactide (pLA_{50}) in $CDCl_3$.

Synthesis of disulfide conjugate 1

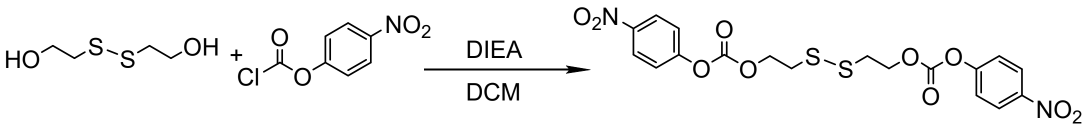

Disulfide conjugate **1** was synthesized *via* a base-catalyzed alcoholysis reaction of 4-nitrophenyl chloroformate and bis(2-hydroxyethyl) disulfide. 4-Nitrophenyl chloroformate (14.69 mmol, 2.96 g) and bis(2-hydroxyethyl) disulfide (7.00 mmol, 1.08 g) dissolved in 6 mL DCM were added into a round-bottom flask, followed by an addition of DIEA (1.40 mmol, 0.18 g). After stirring for 4 h at 45° C, the solvent was removed by evaporation under vacuum and then was washed by 5% citric acid, saturated $NaHCO_3$, and brine. The organic layer was dried over anhydrous Na_2SO_4, filtered, and evaporated under vacuum. The crude residue was purified by flash column chromatography on silica gel (hexane/ethyl acetate=2:1) to afford the conjugate **1** (light yellow solid, 1.6 g, 47.0%).

^{1}H NMR (400 MHz, $CDCl_3$): δ 8.29-8.27 (d, *J* =8, 4H), 7.40-7.38 (d, *J* =8, 4H), 4.59-4.56 (t, *J* =6, 4H), 3.10-3.07 (t, *J* = 6, 4H).

^{13}C NMR (100 MHz, $CDCl_3$): δ 155.4, 152.3, 145.5, 125.4, 121.8, 66.8, 36.8.

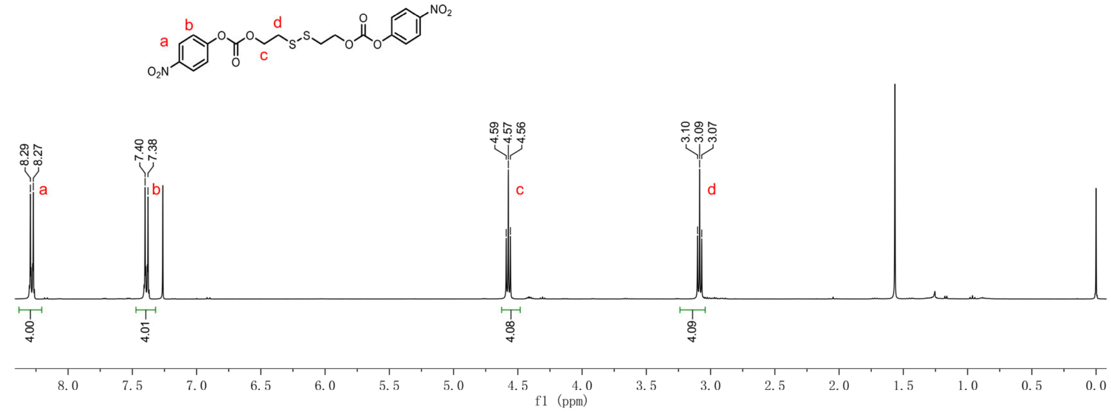

Figure S3. ^1H NMR spectrum of the conjugate **1** in $CDCl_3$.

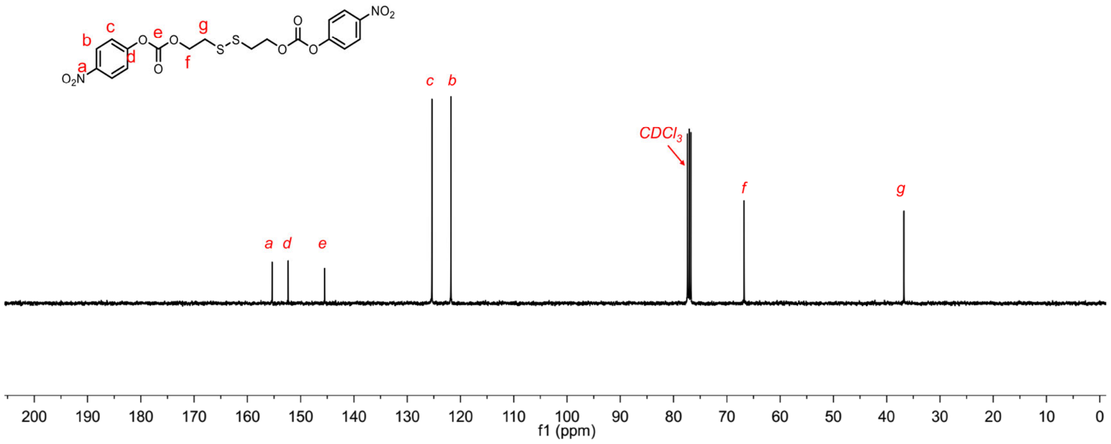

Figure S4. ^{13}C NMR spectrum of the conjugate **1** in $CDCl_3$.

Synthesis of cabazitaxel derivative 2

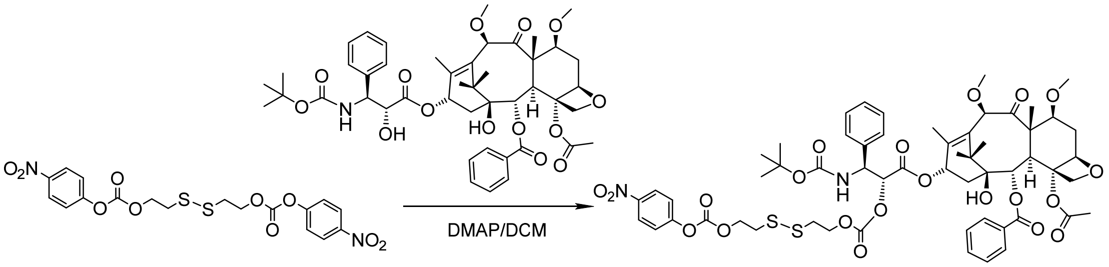

The conjugate **1** (0.50 mmol, 244.14 mg) and 4-dimethylaminopyridine (DMAP, 0.57 mmol, 70.15 mg) were dissolved in 4 mL of DCM. Under stirring at 45° C, a solution of cabazitaxel (0.48 mmol, 400 mg) in 2 mL of DCM was added dropwise into the mixture. After stirring for 30 min, the

residue was immediately purified by flash column chromatography on silica gel (hexane/ethyl acetate=1:1) to afford cabazitaxel derivative **2** (white powder, 227.4 mg, 40.2%).

^{1}H NMR (400 MHz, $CDCl_3$): δ 8.30-8.28 (m, 2H), 8.12-8.10 (d, *J* = 8, 2H), 7.63-7.59 (m, 1H), 7.52-7.48 (t, *J* = 8, 2H), 7.43-7.38 (m, 4H), 7.35-7.32 (m, 3H), 6.31-6.27 (br, 1H), 5.66-5.65 (d, *J* = 4, 1H), 5.47 (s, 2H), 5.27 (s, 1H), 5.01-4.99 (d, *J* = 8, 1H), 4.83 (s, 1H), 4.56-4.49 (m, 2H), 4.44-4.37 (m, 2H), 4.33-4.31 (m, 1H), 4.19-4.17 (m, 1H), 3.92-3.84 (m, 2H), 3.44 (s, 3H), 3.30 (s, 3H), 3.07-2.89 (m, 4H), 2.75-2.67 (m, 1H), 2.44 (s, 3H), 2.33-2.20 (m, 2H), 2.01 (s, 3H), 1.82-1.76 (m, 1H), 1.72 (s, 3H), 1.34 (s, 9H), 1.25 (s, 1H), 1.21 (m, 6H).

^{13}C NMR (100 MHz, $CDCl_3$): δ 205.0, 169.8, 168.1, 167.0, 155.4, 155.2, 154.0, 152.3, 145.5, 139.4, 137.0, 135.2, 133.6, 130.2, 129.3, 129.3, 129.0, 128.7, 128.7, 128.3, 128.3, 126.5, 126.5, 125.4, 125.4, 121.8, 121.8, 84.2, 82.5, 81.6, 80.7, 80.5, 78.9, 76.5, 74.7, 74.7, 72.5, 66.8, 66.6, 57.2, 57.1, 56.8, 47.4, 43.4, 36.8, 36.8, 35.0, 32.0, 29.7, 29.7, 29.7, 28.1, 26.7, 22.9, 21.1, 14.5, 10.4.

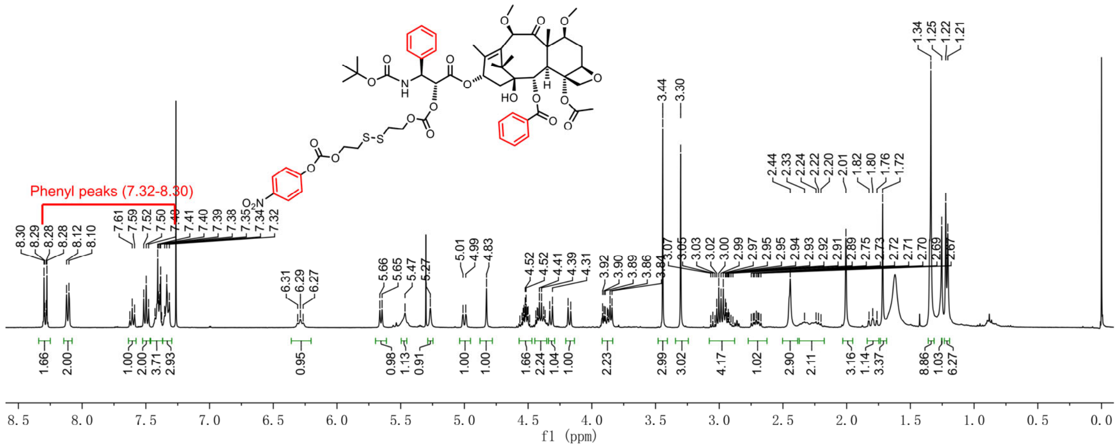

Figure S5. ^{1}H NMR spectrum of cabazitaxel derivative **2** in $CDCl_3$.

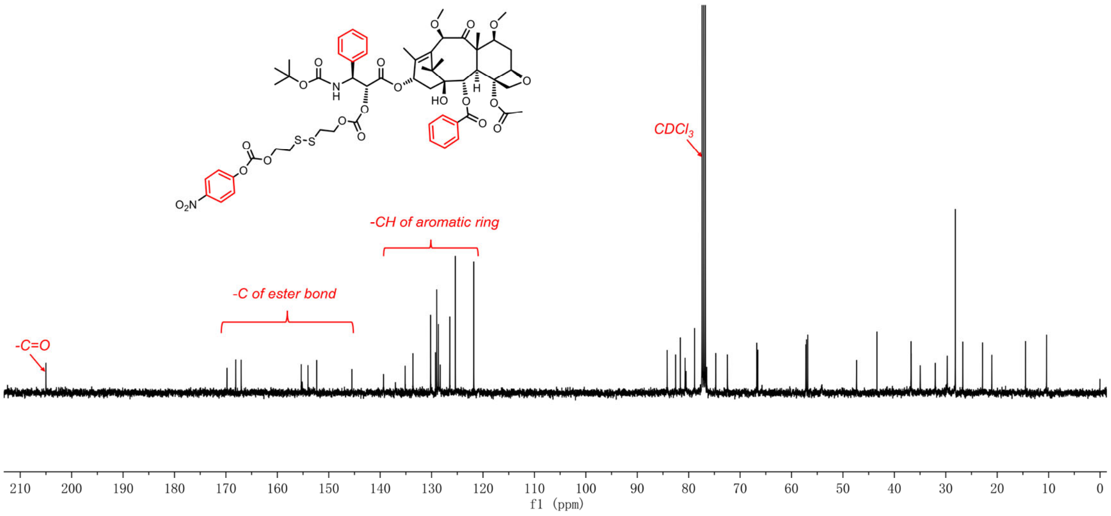

Figure S6. ^{13}C NMR spectrum of cabazitaxel derivative **2** in $CDCl_3$.

Synthesis of pLA$_{15}$-SS-CTX

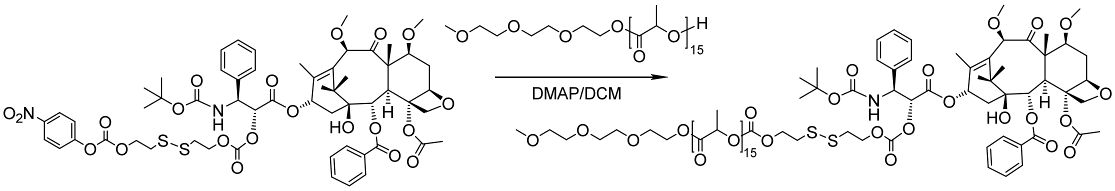

Cabazitaxel derivative **2** (0.19 mmol, 226.81 mg), and pLA$_{15}$-OH (0.16 mmol, 200 mg) were dissolved in 8 mL of DCM and mixed with DMAP (0.19 mmol, 23.21 mg). The reaction mixture was stirred at 45° C for 4 h. Following washing with 5% citric acid, saturated $NaHCO_3$, and brine, the organic layer was dried over anhydrous Na_2SO_4, filtered, and evaporated under vacuum. The residue was purified by flash column chromatography on silica gel (hexane/ethyl acetate=1:2) to afford the pLA$_{15}$-SS-CTX conjugate (white powder, 224.9 mg, 62.0%).

^{1}H NMR (400 MHz, $CDCl_3$): δ 8.12-8.10 (d, 2H, J = 8.0), 7.62-7.59 (m, 1H), 7.52-7.48 (m, 2H), 7.43-7.40 (m, 2H), 7.35-7.32 (m, 3H), 6.31-6.26 (br, 1H), 5.66-5.65 (d, 1H, J = 4.0), 5.47 (s, 1H), 5.26-5.15 (m, 15H), 5.13 (s, 1H), 5.01-4.99 (d, 1H, J = 8.0), 4.83 (s, 1H), 4.39-4.36 (m, 3H), 4.33-4.23 (m, 3H), 4.19-4.16 (m, 1H), 3.92-3.88 (m, 1H), 3.86-3.85 (m, 1H), 3.70-3.68 (m, 2H), 3.64-3.63 (m, 6H), 3.56-3.54 (m, 2H), 3.44 (s, 3H), 3.38 (s, 3H), 3.30 (s, 3H), 2.94-2.90 (m, 3H), 2.75-2.67 (m, 1H) , 2.45 (s, 3H), 2.33-2.20 (m, 2H), 2.01 (s, 3H),

1.82-1.76 (m, 1H), 1.72 (s, 3H), 1.59-1.55 (m, 45H), 1.35 (s, 9H), 1.25 (s, 1H), 1.22-1.21 (m, 6H).

^{13}C NMR (100 MHz, $CDCl_3$): δ 205.0, 170.1-169.2, 168.1, 167.0, 155.2, 154.2, 154.0, 139.5, 137.0, 135.0, 133.6, 130.2, 129.2, 129.2, 129.0, 128.7, 128.7, 128.3, 128.3, 126.5, 126.5, 84.2, 82.5, 81.6, 80.7, 80.5, 78.8, 76.5, 74.7, 74.7, 72.4, 71.9, 71.5, 70.6, 70.6, 69.3-69.0, 68.8, 66.6, 66.0, 64.5, 59.1, 57.2, 57.1, 56.8, 47.3, 43.4, 36.7, 36.6, 36.6, 34.9, 32.0, 29.7, 29.7, 29.7, 28.1, 26.7, 22.9, 21.0, 16.8-16.7, 14.5, 10.4.

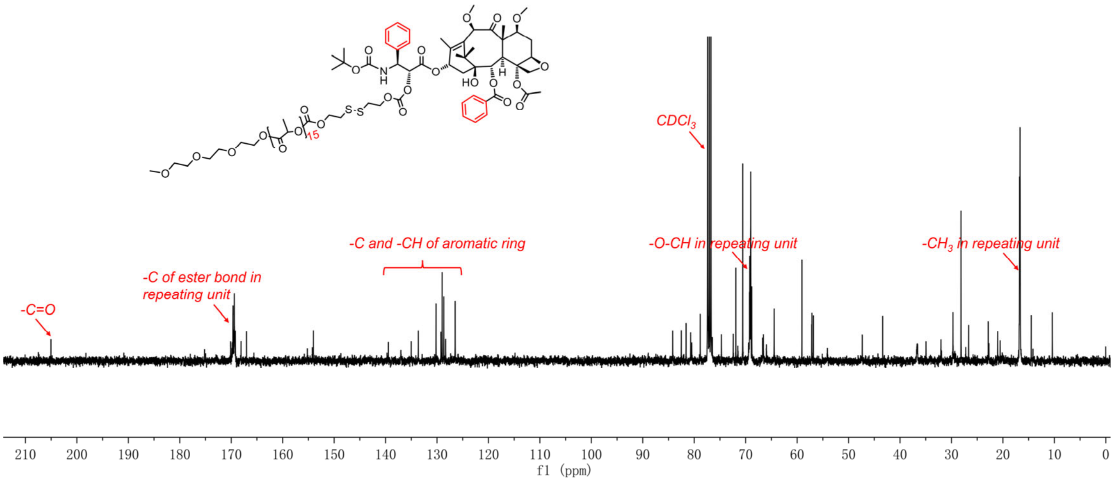

Figure S7. ^{13}C NMR spectrum of pLA_{15}-SS-CTX conjugate in $CDCl_3$.

Synthesis of pLA_{50}-SS-CTX

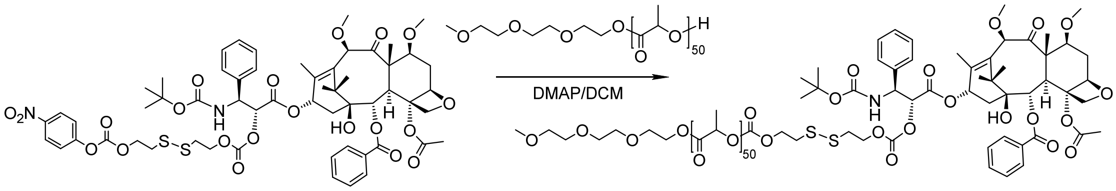

Cabazitaxel derivative **2** (0.13 mmol, 155.93 mg), and pLA_{50}-OH (0.11 mmol, 400 mg) were dissolved in 8 mL of DCM and mixed with DMAP (0.13 mmol, 15.88 mg). The reaction mixture was stirred at 45° C for 4 h. Following washing with 5% citric acid, saturated $NaHCO_3$, and brine, the organic layer was dried over anhydrous Na_2SO_4, filtered, and evaporated under vacuum. The residue was purified by flash column chromatography on silica gel (hexane/ethyl acetate=1:2) to afford pLA_{15}-SS-CTX compound (white powder, 250.0 mg, 47.3%).

^{1}H NMR (400 MHz, $CDCl_3$): δ 8.12-8.10 (d, 2H, J = 8.0), 7.62-7.59 (m, 1H), 7.52-7.48 (m, 2H),7.43-7.40 (m, 2H), 7.34-7.32 (m, 3H), 6.31-6.27 (br, 1H), 5.66-5.65 (d, 1H, J = 4.0), 5.48 (s, 1H), 5.26-5.15 (m, 50H), 5.13 (s, 1H), 5.01-4.99 (d, 1H, J = 8.0), 4.83 (s, 1H), 4.40-4.36 (m, 3H), 4.33-4.26 (m, 3H), 4.19-4.17 (d, 1H, J = 8.0), 3.92-3.88 (m, 1H), 3.86-3.85 (m, 1H), 3.70-3.68 (t, 2H, J = 4.0), 3.65-3.63 (m, 6H), 3.56-3.54 (m, 2H), 3.44 (s, 3H), 3.38 (s, 3H), 3.30 (s, 3H), 2.96-2.93 (m, 3H), 2.75-2.67 (m, 1H), 2.45 (s, 3H), 2.33-2.20 (m, 2H), 2.01 (s, 3H), 1.82-1.76 (m, 1H), 1.72 (s, 3H), 1.59-1.55 (m, 150H), 1.35 (s, 9H), 1.26 (s, 1H), 1.22-1.20 (m, 6H).

^{13}C NMR (100 MHz, $CDCl_3$): δ 205.0, 170.1-169.2, 168.1, 166.9, 155.2, 154.2, 154.0, 139.4, 137.0, 135.0, 133.6, 130.2, 129.3, 129.3, 129.0, 128.7, 128.7, 128.3, 128.3, 126.5, 126.5, 84.1, 82.5, 81.5, 80.7, 80.4, 78.8, 76.4, 74.7, 74.7, 72.4, 71.9, 71.5, 70.5, 70.5, 69.4-69.0, 68.8, 66.6, 66.0, 64.4, 59.0, 57.1, 57.1, 56.8, 47.3, 43.3, 36.7, 36.7, 36.6, 34.9, 32.0, 29.7, 29.7, 29.7, 28.1, 26.6, 22.8, 21.0, 16.7-16.6, 14.5, 10.4.

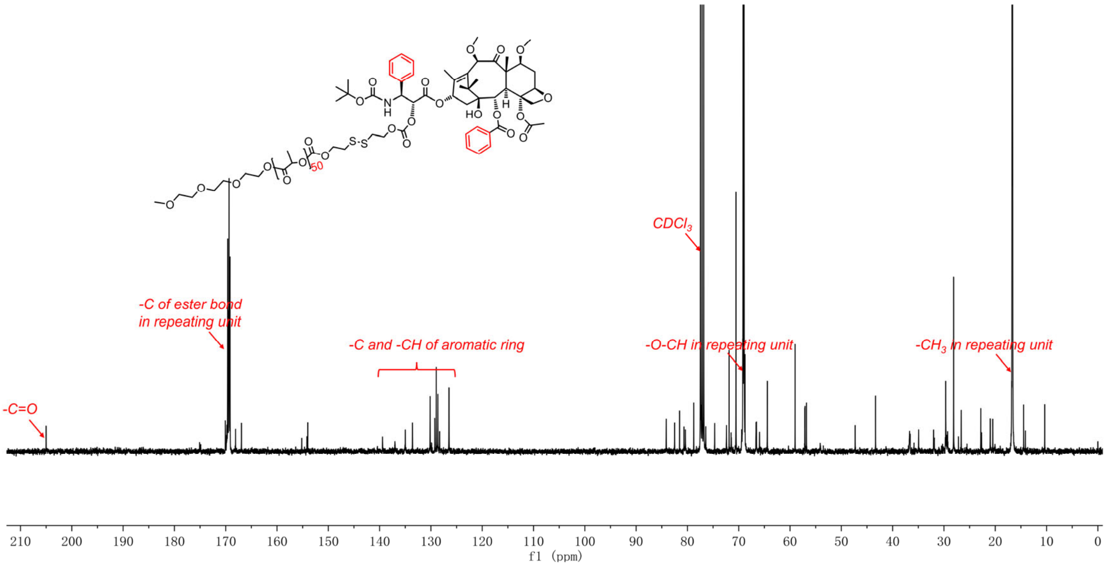

Figure S8. ^{13}C NMR spectrum of pLA_{50}-SS-CTX conjugate in $CDCl_3$.

Analysis of molecular weight distribution

The molecular weight distribution of the pLA_n-SS-CTX conjugates (n = 15 or 50) was analyzed by gel permeation chromatography (GPC) measurement (Waters 1525/2414, USA) at 35° C.

Characterization of pLA_n-SS-CTX NPs.

TEM and SEM analysis

Samples were prepared by dipping pLA_n-SS-CTX NPs (at 0.3 mg/mL cabazitaxel equivalent concentration) onto a 300-mesh copper grid. Following 2 min of deposition, the surface solvent was removed with filter papers and dried at room temperature. Subsequently, samples were positively stained with 2 wt% aqueous uranyl acetate solution for 1 min. Finally, pLA_n-SS-CTX NPs were observed on TECNAL 10 (Philips). For SEM analysis, samples were directly observed with Nova Nano 450 without positive staining.

Dynamic Light Scattering (DLS)

The hydrodynamic diameters (D_H) and distribution and zeta potentials for pLA_n-SS-CTX NPs were measured by a Malvern Nano-ZS90 instrument (Malvern, UK) at 25 ° C.

Determination of drug loading and encapsulation efficiency

The drug loading and encapsulation efficiency of pLA_n-SS-CTX NPs were determined with analytical RP-HPLC. Briefly, pLA_n-SS-CTX NP solutions were centrifuged at 12000 rpm for 10 min to remove the free agents. Subsequently, supernatant was collected and hydrolyzed with 0.1 M NaOH at 37 ° C for 2 h. The final hydrolytic product (i.e., benzoic acid) was used to quantify the amounts of cabazitaxel. The encapsulation efficiency (EE) and drug loading (DL) of cabazitaxel in NPs were calculated as the following formulas:

$$EE\ (\%) = W_{\text{cabazitaxel in NPs}}/W_{\text{feed}} \times 100\% \quad (1)$$

$$DL\ (\%) = W_{\text{cabazitaxel in NPs}}/W_{\text{total}} \times 100\% \quad (2)$$

Evaluation of the stability of pLA_n-SS-CTX NPs

pLA_n-SS-CTX NPs were incubated in DI water and DI water supplemented with 10% FBS (v/v) at 37 ° C. The hydrodynamic diameters (D_H) and zeta potential were monitored over several days.

Determination of critical micelle concentrations (CMCs) for pLA_n-SS-CTX NPs

A series of pLA_n-SS-CTX NP solutions ranging from 1.0×10^{-5} to 1.0 mg/mL were prepared for the measurement of scattering intensity. All samples were detected on a Malvern Nano-ZS90 instrument (Malvern, UK) at 25 ° C. The critical micelle concentrations (CMCs) were determined by plotting the scattering intensity as a function of the logarithm of concentration.

In vitro cytotoxicity assay

The cytotoxicities of pLA_n-SS-CTX NPs against human cancer cell lines including A549, A549/PTX (paclitaxel-resistant cancer cell), HeLa, HeLa/PTX (paclitaxel-resistant cancer cell), DU145 cells and non-cancerous cell lines, including HUVEC and Raw264.7 cells were assessed with CCK8 assay. Cells were seeded in 96-well plates at a density of 1000-3000 cells/well and cultured overnight at 37 ° C. Serial dilutions of free cabazitaxel dissolved in DMSO and pLA_n-SS-CTX NPs were added to each well, respectively. Cells exposed to fresh medium were included as a reference. After 72 h of incubation, the medium for each well was removed and replenished with 100 μL of fresh medium containing 10% CCK8 (v/v). Subsequently, all plates were incubated at 37 ° C for 1-2 h. The absorbance at 450 nm was determined with a microplate reader (Multiskan FC, Thermo Scientific).

EdU assay for assessing cell proliferation

Cell proliferation was evaluated with a Click-iT® EdU Alexa Fluor® 488 Assay Kit (Invitrogen). Briefly, DU145 and HeLa/PTX cells were plated into 48-well plates (2.5×10^4 cells per well for DU145 cells and 5000 cells per well for HeLa/PTX cells) and incubated at 37 ° C for 24 h. Subsequently, free cabazitaxel and pLA_n-SS-CTX NPs were added to cells (2 nM for DU145 cells, 6 nM for HeLa/PTX cells, at cabazitaxel equivalent concentration). After exposure for 48 h, EdU (5-ethynyl-2'-deoxyuridine) was added and incubated for 2 h at 37 ° C. Cells were washed with PBS three times and then fixed with 4% paraformaldehyde for 30 min at room

temperature. Following 20 min of incubation with 0.5% Triton X-100, cells were stained with Alexa Fluor 488 azide for 30 min in the dark and nuclei were stained with Hoechst 33342 for 15 min. Finally, cells were imaged on a fluorescence microscope (Olympus, IX71).

Acridine orange/ethidium bromide (AO/EB) staining assay

The cell apoptosis induced by cabazitaxel formulations was assessed with acridine orange-ethidium bromide (AO/EB) staining assay. DU145 and HeLa/PTX cells were seeded into 48-well plates and cultured for 24 h at 37 ° C. Then, cells were exposed to free CTX and pLA_n-SS-CTX NPs for 48 h (2 nM cabazitaxel-equivalence for DU145 cells, 6 nM cabazitaxel-equivalence for HeLa/PTX cells). The medium for each well was removed and replenished with AO/EB staining solutions premixed at a ratio of 1:1 (v/v). After washing with PBS, the cells were immediately imaged by fluorescence microscopy.

Hemolysis assay

We assessed the hemocompatibility of pLA_{15}-SS-CTX NPs and free cabazitaxel formulated in polysorbate 80 with hemolysis assay. Briefly, fresh rat blood mixed with anticoagulant (EDTA) was centrifuged at 1500 rpm for 10 min to isolate red blood cells (RBCs). Upon complete washes with saline, 300 μL of RBCs suspension (2% RBSs, v/v) was incubated with the same volume of pLA_{15}-SS-CTX NPs and free cabazitaxel formulated in polysorbate 80 at predetermined concentrations (0.5, 1, 1.5, 2, and 3 mg/mL) at 37 ° C for 1 h. Saline and 1% Triton X-100 were included as the negative and positive controls, respectively. Subsequently, intact RBCs were isolated by centrifugation at 1500 rpm for 10 min, while the supernatant for each group was transferred into 96-well plates. Absorbance at 540 nm was measured on a microplate reader (Multiskan FC, Thermo Scientific). The hemolysis was calculated using the following formula:

$$Hemolysis\ (\%) = 100\% \times (A_{\mathrm{samples}} - A_{\mathrm{saline}}) / (A_{\mathrm{Triton}} - A_{\mathrm{saline}})$$

Animal studies

Mice (5-6 weeks old) for animal studies were purchased from Shanghai Experimental Animal Centre, Chinese Academy of Science. All animal experiments were performed in compliance with the guidelines of the Zhejiang University Committee for Animal Use and Care. The studies involving animals were approved by the Ethics Committee of the First Affiliated Hospital, Zhejiang University School of Medicine. They were housed under aseptic conditions and given an autoclaved rodent diet and sterile water.

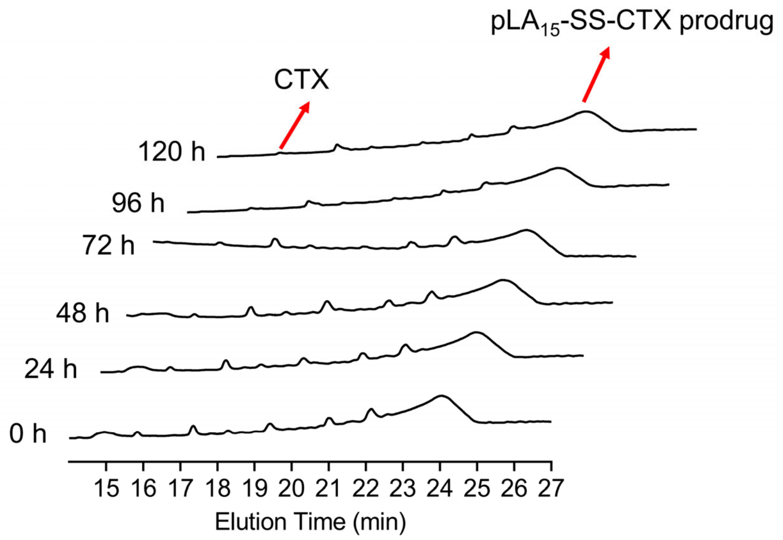

Figure S9. Representative HPLC chromatograms of pLA_{15}-SS-CTX prodrug in the absence of DTT.

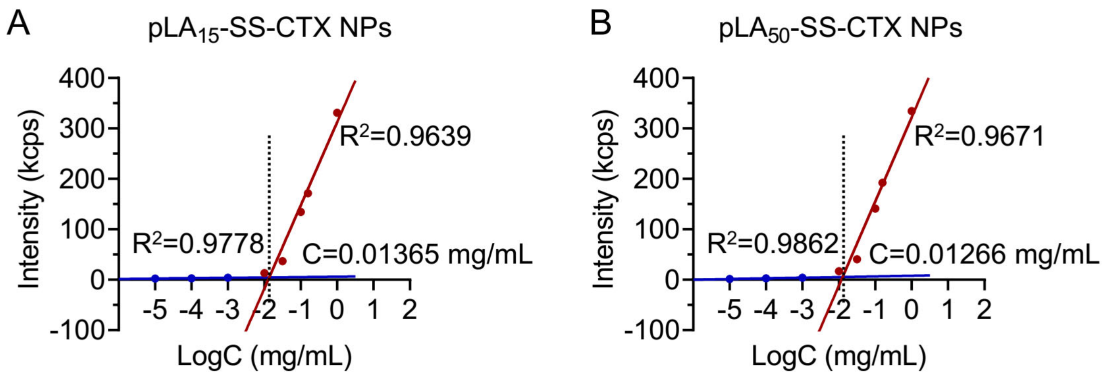

Figure S10. Determination for the critical micelle concentration (CMC) of pLA_n-SS-CTX NPs by measuring the intensity of scattered light (kcps).

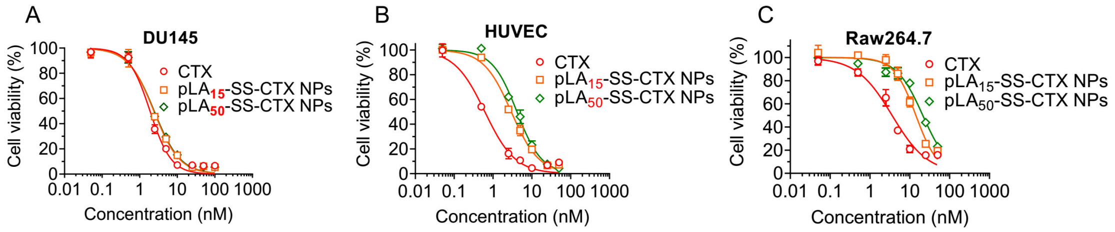

Figure S11. *In vitro* cytotoxicity of free cabazitaxel and pLA$_n$-SS-CTX NPs (n = 15 or 50) against **A**) human prostate cancer cell (DU145) and **B** and **C**) normal cells (HUVEC and Raw 264.7).

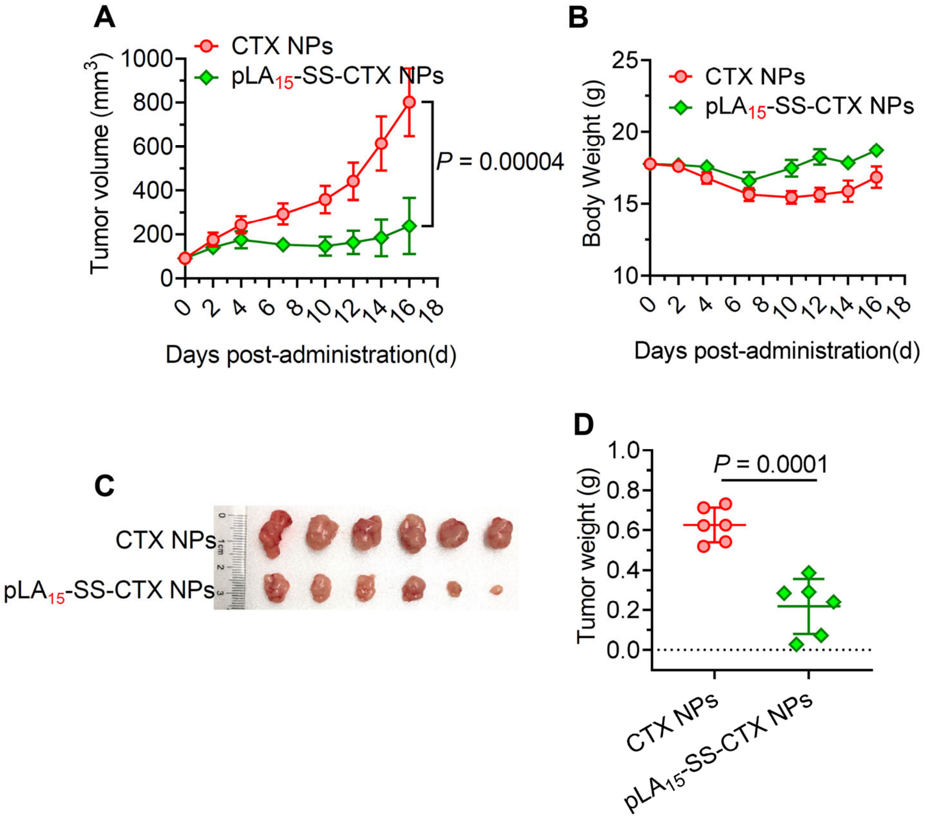

Figure S12. *In vivo* efficacy studies of pLA$_{15}$-SS-CTX NPs against paclitaxel-resistant HeLa/PTX cervical tumor xenografts. Mice were intravenously injected with cabazitaxel-loaded NPs (CTX NPs) and pLA$_{15}$-SS-CTX NPs at a cabazitaxel-equivalent dose of 6 mg/kg for three times. **A**) Tumor growth curves upon drug treatment. **B**) Variations in body weight following drug administration. **C**) Photograph of the tumors excised from each treatment group at the endpoint of the study. **D** Tumor weight in each group.

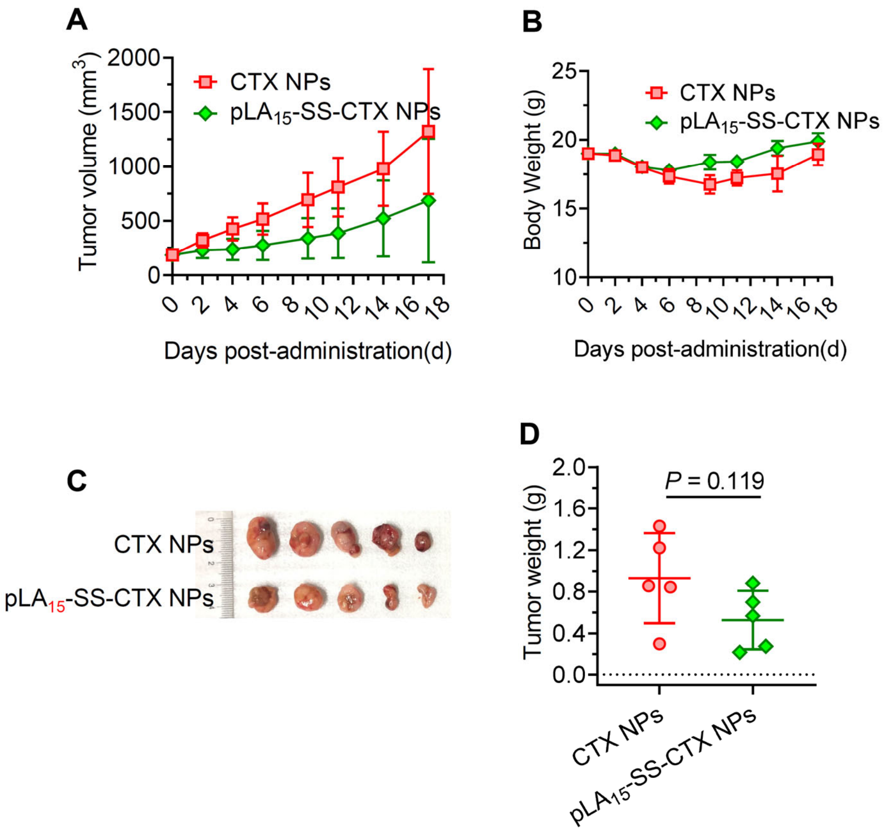

Figure S13. *In vivo* efficacy studies of pLA_{15}-SS-CTX NPs against paclitaxel-resistant A549/PTX lung tumor xenografts. Mice were intravenously injected with cabazitaxel-loaded NPs (CTX NPs) and pLA_{15}-SS-CTX NPs at a cabazitaxel-equivalent dose of 6 mg/kg for three times. **A**) Tumor growth curves upon drug treatment. **B**) Variations in body weight following drug administration. **C**) Photograph of the tumors excised from each treatment group at the endpoint of the study. **D**) Tumor weight in each group.

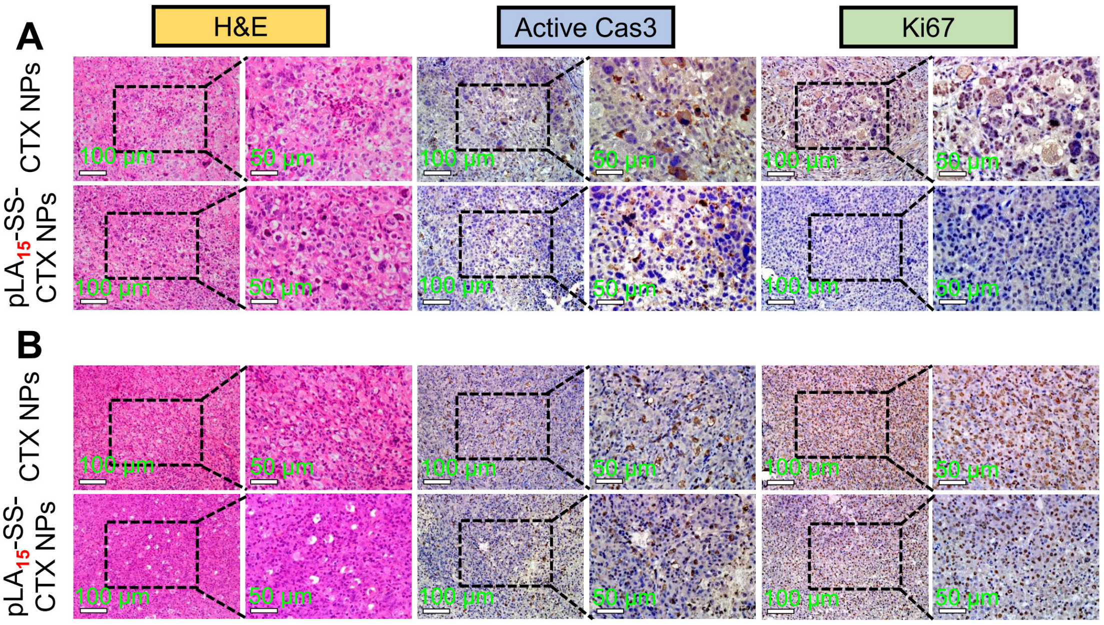

Figure S14. H&E, active (cleaved) caspase-3, and Ki67 staining of **A**) HeLa/PTX and **B**) A549/PTX tumor sections at 10 d postadministration.

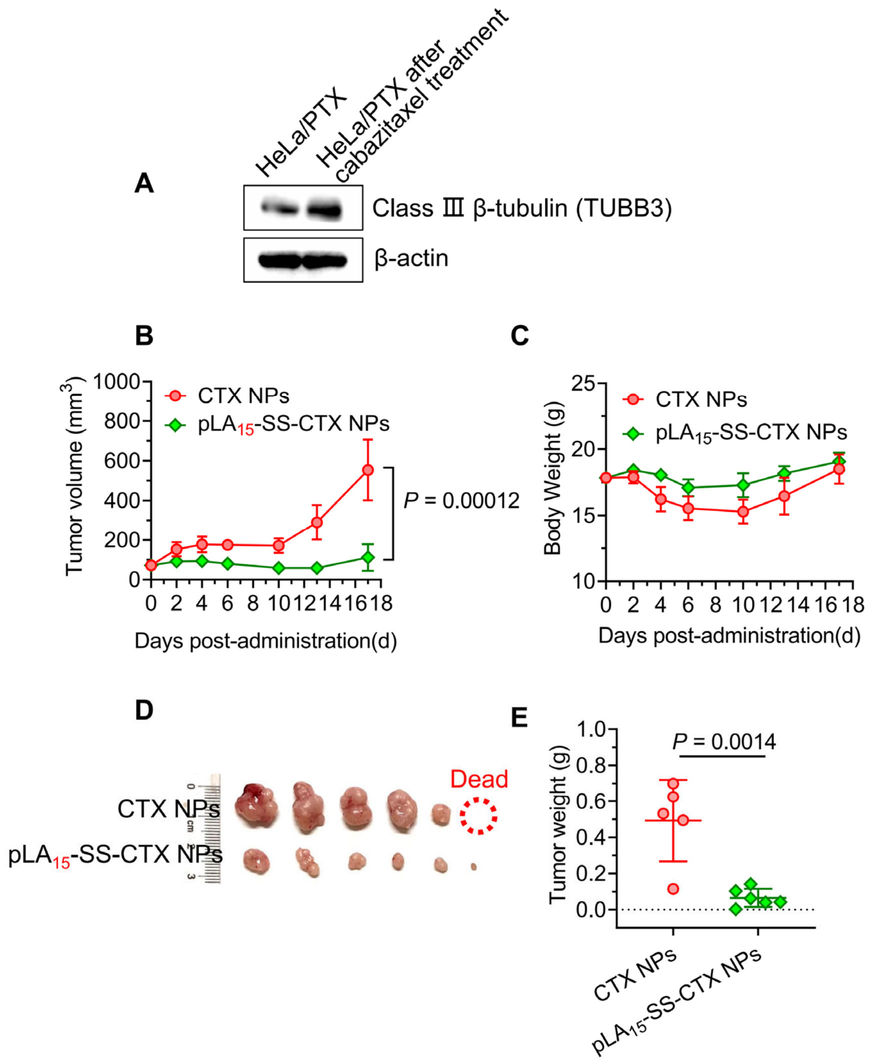

Figure S15. *In vivo* efficacy studies of pLA$_{15}$-SS-CTX NPs against recurrent tumors. HeLa/CTX cervical tumors were pretreated with free cabazitaxel (6 mg/kg) every three days for six times, and subsequently, the tumors were re-transplanted into Balb/c nude mice. When the recurrent tumor volumes reached ~70 mm^3, mice were intravenously injected with cabazitaxel-loaded NPs (CTX NPs) and pLA$_{15}$-SS-CTX NPs at a cabazitaxel-equivalent dose of 6 mg/kg for three times. **A**) Expression of the class III (TUBB3) β-tubulin isotype evaluated with western blot from HeLa/PTX tumors and HeLa/CTX tumors pretreated with free cabazitaxel (6 mg/kg) for six times. **B**) Tumor growth curves upon drug treatment. **C**) Variations in body weight following drug administration. **D**) Photograph of the tumors excised from each treatment group at the endpoint of the study. **E)** Tumor weight in each group.
